# Tobacco-specific nitrosamine exposure from electronic cigarettes versus combustible cigarettes: an *ad hoc* analysis within a systematic review of emission studies

**DOI:** 10.3389/fonc.2025.1729107

**Published:** 2025-12-12

**Authors:** Red Thaddeus D. Miguel, Rami Ali, Manal El Joumaa

**Affiliations:** Thera-Business Inc., Ottawa, ON, Canada

**Keywords:** TSNA, tobacco-specific nitrosamines, NNK, NNN, E-cigarettes, cigarette smoke, carcinogen exposure, harm reduction

## Abstract

**Introduction:**

Tobacco-specific nitrosamines (TSNAs), particularly 4-(methylnitrosamino)-1-(3-pyridyl)-1-butanone (NNK) and N′-nitrosonornicotine (NNN), have been shown to be among the most potent carcinogens found in tobacco products. With the rapid adoption of electronic cigarettes (e-cigarettes) as alternatives to combustible cigarettes, understanding the extent of TSNA exposure has become central to oncology practice and risk communication.

**Methods:**

This ad hoc analysis, based on studies identified from a systematic review of e-cigarette emissions, synthesized evidence from 13 emission studies that directly compared NNK and NNN levels between e-cigarette aerosols and cigarette smoke. Eligible studies were identified through comprehensive database searches (MEDLINE, Embase, and ToxFile) and assessed for methodological rigor using an adapted QualSyst framework.

**Results:**

Across studies, validated analytical methods, primarily LCMS/MS and UPLC-MS, demonstrated that TSNAs in e-cigarette aerosols were either undetectable or present at concentrations lower than those in combustible cigarette smoke, with reductions typically exceeding 99%. The findings show a toxicological difference between combustible cigarettes and e-cigarettes, with the latter exhibiting substantially reduced TSNA emissions comparable to laboratory background air levels.

**Discussion:**

These results suggest that switching to exclusive e-cigarette use can lead to a significant reduction in exposure to key tobacco-specific nitrosamines. This study also reinforces the importance of articulating this evidence with clarity, precision, and balance, recognizing both the substantial benefits of reduced exposure and the residual uncertainties that only long-term studies will resolve.

## 1 Introduction

Tobacco-specific nitrosamines (TSNAs) are a group of carcinogenic compounds formed primarily during the curing and processing of tobacco. Among these, 4-(methylnitrosamino)-1-(3-pyridyl)-1-butanone (NNK) and N’-nitrosonornicotine (NNN) are the most biologically significant as they are both classified by the International Agency for Research on Cancer (IARC) as Group 1 carcinogens (1). They are also listed as Harmful and Potentially Harmful Constituents (HPHCs) in tobacco products and tobacco smoke by the FDA (2). Their carcinogenicity is mediated through cytochrome P450-driven α-hydroxylation, leading to DNA adduct formation and subsequent mutations in oncogenes and tumor suppressor genes (3). Substantial evidence from animal models and human epidemiological studies links NNK to lung adenocarcinoma (4, 5) and NNN to esophageal squamous cell carcinoma (6). Collectively, exposure to TSNAs represents a well-established biological pathway to carcinogenesis, rendering their quantification a benchmark for assessing the carcinogenic risk of tobacco and nicotine products.

Over the past decade, electronic cigarettes (e-cigarettes) have emerged as a crucial nicotine delivery system within the field of harm reduction. Their rapid popularity and widespread adoption are largely driven by the ability to deliver nicotine without combustion coupled with a perception of reduced harm compared to conventional cigarettes (7, 8). Consequently, e-cigarettes are increasingly used for both smoking cessation and recreational purposes (9). A Cochrane systematic review led by Lindson (10) demonstrated that nicotine-containing e-cigarettes are associated with increased quit rates compared to conventional nicotine replacement therapies (NRT) or e-cigarettes without nicotine. Evidence from 90 completed studies, including 49 randomized controlled trials, suggests that nicotine e-cigarettes are more effective than nicotine replacement therapy for smoking cessation, with quit rates about 1.6 times higher (RR 1.59, 95% CI 1.30–1.93) (10). Overall, adverse events were generally mild and comparable across groups, while serious adverse events were rare and too infrequent to establish clear differences (10). While these findings support the potential of e-cigarettes as a cessation aid, concerns persist regarding their long-term safety, particularly with regard to user exposure to potentially toxic substances present in their emissions (9, 10). These concerns are further complicated by the heterogeneity of e-cigarette devices, evolving formulations, and their dual use with combustible cigarettes (11).

Despite the absence of combustion, the extent of TSNA exposure from e-cigarette use remains a topic of ongoing investigation and debate. Several studies demonstrate that e-cigarettes produce notably lower levels of toxicants than combustible cigarettes; however, evidence specific to TSNA emissions from e-cigarettes is inconsistent. Some studies reported undetectable levels of TSNAs in e-cigarette aerosols, while others identified measurable levels. Regardless of the detected or undetected levels of TSNAs in e-cigarette emissions, evidence consistently shows that cigarette smoke produces higher TSNA levels than e-cigarette aerosols. Additionally, some investigations, conducted without direct cigarette comparators, suggested the presence of TSNAs across different e-cigarette products, with concentrations influenced by device design, heating mechanisms, and formulation characteristics.

Despite the absence of combustion, the degree to which e-cigarette use contributes to TSNA exposure remains uncertain and contested. While several studies suggest that e-cigarettes emit substantially lower levels of toxicants than combustible cigarettes, the evidence specific to TSNAs is heterogeneous. Some analyses have reported TSNAs to be undetectable in e-cigarette aerosols (12, 13), whereas others have detected measurable and at times variable concentrations (14). Moreover, although comparative data generally show cigarette smoke yielding higher TSNA levels than e-cigarette emissions, methodological differences in aerosol collection, analytical sensitivity, and device selection complicate interpretation across studies (15). Additional investigations conducted without direct cigarette comparators have further highlighted product-dependent variability, with TSNA concentrations appearing to differ according to device type, heating parameters, and liquid composition (14, 16). Taken together, these inconsistencies underscore the need for a systematic evaluation of the available evidence to clarify the extent and determinants of TSNA exposure from e-cigarette use.

Fragmented and conflicting data have perpetuated uncertainty, and patients increasingly seek guidance on whether e-cigarettes reduce cancer risk compared with traditional smoking, emphasizing the need for clear, evidence-based communication. The purpose of this analysis was to address this gap by systematically comparing TSNA levels reported in e-cigarette emissions and combustible cigarette smoke. By synthesizing the available data, the goal was to provide a definitive and practical understanding of relative TSNA exposure, ensuring that cancer risk communication is grounded in toxicological evidence.

## 2 Methods

This synthesis was undertaken as an analysis of comparative studies from the search results of a systematic review of e-cigarette emissions, with a focus on TSNAs. The primary research question was:

What are the levels of NNK and NNN detected in e-cigarette aerosols compared with combustible cigarette smoke under consumer-relevant laboratory conditions?

### 2.1 Data sources and searches

This was an *ad hoc* analysis from studies identified from the search results of a systematic review of e-cigarette emissions (PROSPERO registration CRD420251159693) (17). The systematic review addressed the question: in laboratory-based physicochemical emission studies that assess e-cigarette aerosols, what are the levels of carbonyls, metals, NNK, and NNN detected under consumer-relevant usage conditions? That review evaluated a broad range of HPHCs, including carbonyls, metals, and TSNAs, and was guided by the AMSTAR 2 (A Critical Appraisal Tool for Systematic Reviews) (18) guidelines and the PRISMA (Preferred Reporting Items for Systematic Review and Meta-Analysis) statement (19).

The breadth of the systematic review outlined above permitted the identification and isolation of a subset of studies that provided direct comparisons of NNK and NNN levels in e-cigarette aerosols and in cigarette smoke. The present analysis focused on these studies to address a persistent gap in the cancer literature, where such comparisons have not previously been systematically synthesized despite their central importance to carcinogenesis. The objective of this analysis was to characterize the consistency, magnitude, and credibility of observed differences between e-cigarettes and cigarettes in relation to these nitrosamines.

The evidence base for this analysis was derived from the comprehensive searches performed for the systematic review. During the scoping phase, previously published systematic reviews and original articles were reviewed to identify evidence gaps and inform the development of the search strategy. The search strategy was developed with support from subject matter experts to maximize sensitivity for laboratory-based emission studies while retaining specificity for toxicological outcomes of interest. Controlled vocabulary terms were combined with free-text keywords for e-cigarettes and with terms relating to carbonyls, metals, and TSNAs. Chemical synonyms and CAS registry numbers were incorporated to ensure capture of studies that did not employ standard nomenclature. Searches were conducted in MEDLINE, Embase, and ToxFile, covering records from inception through July 10, 2025. The search strategy extended beyond databases. Reference lists of all included articles and of relevant systematic reviews were hand-searched, and authoritative reports were examined for additional citations.

Only full-text, peer-reviewed publications in English reporting original data were eligible. The comprehensive scope of the systematic review used to identify studies for this analysis ensured identification of a large and diverse set of emission studies across devices, puffing regimens, and analytical methods. From this body of work, the subset of studies reporting side-by-side measurements of NNK and NNN in e-cigarette aerosols and combustible cigarette smoke formed the foundation of the present analysis.

### 2.2 Eligibility criteria

Eligible studies were laboratory-based physicochemical investigations of e-cigarette aerosol emissions. To be considered, studies had to generate aerosol from nicotine-containing or nicotine-free e-cigarette products under conditions designed to reflect consumer use. Devices had to rely on heating elements for aerosolization, and methods had to specify the type of device tested and the puffing regimen employed.

Eligible studies quantified levels of NNK or NNN in both e-cigarette aerosol and combustible cigarette smoke, using validated analytical techniques. Emissions were required to be reported in absolute values and standardized units, such as per puff, per milligram of aerosol, or per cubic meter of air, to allow comparison across studies. Limits of detection (LOD) and quantification (LOQ) were also noted when provided by the study, as they offered critical context for interpreting non-detectable or low-level findings.

Studies were excluded if they did not report quantitative emissions of NNK or NNN in aerosols, if they assessed only e-liquids or device components without aerosol generation, or if they focused exclusively on combustible cigarettes, heated tobacco products, or cannabis-containing devices. Studies relying on unrealistic or artefactual conditions, such as protocols producing known dry puffing without justification or applying puffing regimens implausible for the device evaluated, were also excluded, as were reports that provided only relative values without corresponding absolute measurements or that lacked evidence of background controls such as air blanks. Research limited to biomarkers of exposure, toxicological assays, animal or ex vivo models, or in silico simulations was not eligible. Reviews, commentaries, conference abstracts, government reports, and other non–peer-reviewed sources were excluded, as were duplicate publications and non-English articles. These criteria ensured that the evidence base comprised rigorously conducted laboratory studies capable of informing a direct comparison of NNK or NNN emissions between e-cigarettes and combustible cigarettes.

### 2.3 Study selection

Records identified through the searches underwent two-stage screening. Titles and abstracts were reviewed to exclude clearly ineligible reports only, with decisions coded as exclude or carry forward. No study was included based on title and abstract alone. All records not excluded at this stage proceeded to full-text assessment. At both stages, two reviewers evaluated each record independently using predefined eligibility criteria and a standardized screening form following a brief calibration exercise.

Agreement between reviewers was tracked across all dual assessments. Discrepancies were resolved by discussion to consensus; unresolved disagreements were adjudicated by a third reviewer. Reasons for exclusion at the full-text stage were documented to ensure transparency and reproducibility.

### 2.4 Data extraction and management

Data extraction was conducted by one research associate and checked independently by a second, with disagreements resolved through discussion or by referral to a third team member where necessary. A pre-specified extraction form was used to ensure consistency across studies. For each eligible study, information was captured on study design, setting, and funding source, along with detailed descriptions of the devices evaluated, the puffing regimens employed, and the laboratory procedures used to generate and collect aerosol. Particular attention was paid to parameters that might influence emission levels, including device brand and model, heating element characteristics, power settings, and e-liquid composition. Data on the number of devices, replicates, and samples analyzed were also recorded, as were details of the analytical methods, including collection media and detection limits.

Quantitative results for NNK and NNN were extracted in the original units reported. Both outcome data and information relevant to interpreting study quality and methodological rigor were documented to support subsequent synthesis. All records were managed in Rayyan, a web-based systematic review platform.

### 2.5 Quality assessment

Study quality was assessed using the QualSyst tool for systematic reviews of quantitative studies (20). This tool, which synthesizes elements from several validated instruments, evaluates core aspects of design, methodology, analysis, and reporting. Following the approach described by Zhao et al. (21), the instrument was adapted to reflect the specific requirements of e-cigarette emission research. The adapted tool included 21 criteria, extending beyond the original 14 to capture key elements such as reporting of device and e-liquid characteristics, adequacy of sample size, use of blanks and background correction, laboratory quality control procedures, specification of detection limits, appropriateness of puffing regimens to device type, and transparency in aerosol collection and analytical methods. These additions were considered essential to evaluate the internal validity of studies and to interpret their toxicological implications.

Each criterion was scored on a three-point scale, with 2 assigned when the criterion was fully met, 1 when partially met, and 0 when not met or not reported. Items judged not applicable to a study design were excluded from the denominator in calculating the overall score. Quality scores were expressed as the ratio of points achieved to the maximum possible score, permitting standardized comparison across studies. A provisional quality threshold between 0.55 and 0.75 was applied. Discrepancies in scoring between reviewers were resolved by discussion, and when agreement could not be reached, a third reviewer adjudicated the final decision.

### 2.6 Analysis

Findings from the included studies were synthesized narratively. The synthesis was structured around device type, usage conditions, analytical methods, and outcome measures, with attention to consistency and direction of results. The original protocol anticipated that, where sufficient homogeneity existed across studies, results would be pooled using a random-effects meta-analysis. This was not feasible, as reporting formats varied considerably across studies and precluded calculation of standardized effect measures. Instead, results are presented descriptively, with emphasis on the observed magnitude and reproducibility of differences between e-cigarette aerosols and cigarette smoke.

Sensitivity and subgroup analyses were also pre-specified. These included plans to examine the influence of methodological quality, device type, heating element resistance and temperature, power output, puffing regimens, and analytical techniques. Given the heterogeneity of reporting and the limited number of directly comparable studies, these analyses could not be performed quantitatively. Instead, narrative comparisons were used to highlight methodological features that may contribute to differences in reported outcomes.

## 3 Results

### 3.1 Characteristics of included studies

A total of 1,633 articles were retrieved from the specified databases used to search for eligible articles in the referenced systematic review. For the current *ad hoc* analysis, 1,622 studies were excluded, resulting in 11 relevant studies eligible for inclusion (14, 16, 22–30). Two additional papers from snowballing methods were also identified to have met the inclusion criteria of this analysis (31, 32). **Table 1** summarizes the main characteristics of the 13 included studies. A more detailed description of study and device characteristics, experimental conditions, and outcomes in each study is provided in **Supplementary Material** (**Table 1**).

**Table 1:** Summary of device type, methodology, and study characteristics.

Author	Country	Industry-funded	EC device type	EC device description	liquid/flavor	Number of samples	Puffing topography method	Analytical method	Risk of bias^*^
Chen et al., 2021 (31)	US	Yes (All authors employed by Juul Labs)	EC01: Pod	EC01 Brand: JUUL pod	EC01: Nic: 3% and 5%; Flavors: Virginia Tobacco and Menthol	30 (10 replicates of 3 JUUL flavor/concentration for each regimen)	Non-Intense samples: ISO 20768 (Dur: 3 sec; Int: 30 sec; Vol: 55 mL; Puffs: 50) Intense samples: (Dur: 6 sec; Int: 30 sec; Vol: 110 mL; Puffs: 50)	LC-MS/MS (ESI)	S
Cook et al., 2024 (22)	US	Yes (All authors employed by Juul Labs; Testing and analytics were funded by Juul Labs)	EC01: Pod	EC01 Brand: JUUL2 System; consisted of a closed pod and technologically advanced (smartphone-compatible) device	EC01: Nic: 18mg/mL; Flavors: Virginia Tobacco, Crisp Menthol, Polar Menthol, Autumn Tobacco, Ruby Menthol, and Summer Menthol	6 formulations	Non-Intense samples: ISO 20768:2018 (Dur: 3 sec; Int: 30 sec; Vol: 55 mL; Puffs: 100-150; Sets: 2) Intense samples: ISO 20768:2018 (Dur: 6 sec; Int: 30 sec; Vol: 110 mL; Puffs: 60-90; Sets: 2)	GC-MS/MS	S
Cunningham et al., 2020 (23)	UK	Yes (BAT)	EC01 & EC02: Closed system	EC01 Brand: Vype, ePen2; consisted of a reusable section containing a rechargeable battery and disposable flavor cartridge (Watt: 4.4W; Rechargeable battery 650 mAh) EC02 Brand: Vype, ePen3; contained protective battery electronics that limit dry-puff events (NiFe Alloy; Res: 1.95-2.36Ω; Watt: 5.9W; Rechargeable battery 650 mAh)	EC01: Nic: 18mg/mL; Flavor: Blended Tobacco; PG/VG: 25.00%/48.22% (w/w) EC02: Nic: 12, 18, and 30mg/mL; Flavors: ePen3 18: Blended Tobacco; ePen3–12 Low BA, ePen3–18 Medium BA, and ePen3–30 High BA: MasterBlend; PG/VG: ePen3 BT 18: 54.00%/34.22% (w/w); ePen3 MB 12 Low BA: 54.25%/34.57% (w/w); ePen3 MB 18 Medium BA: 54.73%/33.5% (w/w); ePen3 MB 30 High BA: 56.06%/31.2% (w/w)	5 EC variants	CORESTA No.81 (ISO 20768) (Dur: 3 sec; Int: 30 sec; Vol: 55 mL; Puffs: 50; Sets: 1)	LC-MS	S
Goniewicz et al., 2014 (14)	Poland & US	No (MEIN and NIH)	EC01-12: Cig-a-like	EC01 Brand: Joye510 EC02 Brand: Janty eGo EC03 Brand: Janty Dura EC04 Brand: DSE 910 EC05 Brand: Trendy 808 EC06 Brand: Nicore M401 EC07 Brand: Mild 201 EC08 Brand: Colinss Age EC09 Brand: Premium PR111 EC10 Brand: Ecis 510 EC11 Brand: Dekang Pen EC12 Brand: Intellicig Evolution	EC01: Nic: 4mg/mL; Flavor: Marlboro EC02: Nic: 16mg/mL; Flavor: Marlboro EC03: Nic: 16mg/mL; Flavor: Marlboro EC04: Nic: 16mg/mL; Flavor: Regular EC05: Nic: 18mg/mL; Flavor: Trendy EC06: Nic: 18mg/mL; Flavor: Marlboro EC07: Nic: 18mg/mL; Flavor: Marlboro EC08: Nic: 18mg/mL; Flavor: Camel EC09: Nic: 16mg/mL; Flavor: Tobacco EC10: Nic: 11mg/mL; Flavor: Menthol EC11: Nic: 18mg/mL; Flavor: Regular EC12: Nic: 8mg/mL; Flavor: Regular	NR	Based on results of inhalation topography measurement among 10 regular EC users (Dur: 1.8 sec; Int: 10 sec; Vol: 70 mL; Puffs: 150; Sets: 10 of 15 puffs)	UPLC-MS	G
Jin et al., 2022 (16)	US	Yes (All authors were employed by Altria; Methods developed and validated internally at Altria)	EC01-08: Cig-a-like	EC01 Brand: NR (Res: 3.4Ω) EC02 Brand: NR (Res: 3.4Ω) EC03 Brand: NR (Res: 3.4Ω) EC04 Brand: MarkTenXL (Res: 3.6Ω) EC05 Brand: MarkTenXL (Res: 3.5Ω) EC06 Brand: NR (Res: 2.8Ω) EC07 Brand: NR (Res: 2.3Ω) EC08 Brand: NR (Res: 2.1Ω)	EC01: Nic: 2.14%; PG/VG: 33.4%/53.6% (w/w) EC02: Nic: 1.7%; PG/VG: ND/85.2% (w/w) EC03: Nic: 2.17%; PG/VG: 35.0%/54.2% (w/w) EC04: Nic: 3.34%; Flavor: Menthol; PG/VG: 49.6%/33.2% (w/w) EC05: Nic: 3.36%; Flavor: Classic; PG/VG: 23.8%/56.1% (w/w) EC06: Nic: 4.56%; PG/VG: 16.9%/66.8% (w/w) EC07: Nic: 4.43%; Flavor: Menthol; PG/VG: 24.2%/59.0% (w/w) EC08: Nic: 4.33%; PG/VG: 24.1%/59.2% (w/w)	NR	Similar to CORESTA Method No. 81 (Dur: 5 sec; Int: 30 sec; Vol: 55 mL; Puffs: 150 (except for 50 puffs for MarkTen XL in the experiment of nitrite fortification to e-liquid; Sets: 1)	UPLC-MS/MS	G
Leigh et al., 2018 (32)	US	No (NCI of the NIH)	EC01: Cig-a-like	EC01 Brand: MarkTen	EC01: Nic: 3.5%; Flavor: Tobacco	NR	Health Canada Intense (Dur: 2 sec; Int: 30 sec; Vol: 55 mL; Puffs: 55)	LC-MS/MS	A
Margham et al., 2016 (25)	UK	Yes (BAT)	EC01: Closed-modular system	EC01 Brand: Vype, ePen; consisted of rechargeable battery section and replaceable e-liquid containing cartridge (Volt: 3.6V; NiCr Alloy; Res: 2.85Ω; USB-Rechargeable battery 650 mAh)	EC01: Nic: 1.86%; Flavor: Blended Tobacco; PG/VG: 25%/48.14% (w/w)	1 product was sampled at a single point in time	CORESTA No.81 (Dur: 3 sec; Int: 2/min; Vol: 55 cm^3^; Puffs: 200; Sets: 2 of 100 puffs each)	LC-MS/MS	S
Margham et al., 2021 (24)	UK	Yes (BAT)	EC01: Closed system	EC01 Brand: Vype, ePen2; consisted of rechargeable battery and disposable e-liquid cartridge (Volt: 3.5-3.7V; NiCr Alloy; Res: 2.85Ω; Micro-USB rechargeable battery)	EC01: Nic: 1.86%; Flavors: Golden Tobacco, Dark Cherry, and Crisp Mint; PG/VG: Golden Tobacco: 24.97%/48.14% (w/w); Dark Cherry: 23.86%/48.14% (w/w); Crisp Mint: 34.73%/37.64% (w/w)	3 flavor variants of the EC	CORESTA No.81 (ISO 20768:2018) (Dur: 3 sec; Int: 2/min; Vol: 55 mL; Puffs: 100; Sets: 2 of 100 puffs each)	LC-MS/MS	S
Nicol et al., 2020 (26)	UK	Yes (BAT)	EC01: ePen	EC01 Brand: NR; consisted of rechargeable battery and disposable cartridge (Stainless Steel Mesh; Watt: 10W; Rechargeable battery)	EC01: Nic: 5mg/mL; Flavor: Twilight Tobacco; PG/VG: 36%/62.6% (w/w)	1 flavor variant sampled from the factory at a single point in time	CORESTA No.81 (ISO 20768:2018) (Dur: 3 sec; Int: 30 sec; Vol: 55 mL; Puffs: 50; Sets: 1)	LC-MS/MS	S
Pinto et al., 2022 (27)	UK	Yes (BAT)	EC01: Pod	EC01 Brand: Vype, ePod1.0; consisted of a lithium-ion rechargeable battery, a cartridge, and each pod was pre-filled with Vype e-liquid (Volt: 2.2-3.1V; NiCr Alloy; Res: 0.8-1.4Ω; Watt: 6.5 ± 0.5W; Lithium-ion rechargeable battery 350 mAh)	EC01: Nic: 18 and 57mg/mL; Flavor: Berry Blast; PG/VG: 50%/50% (w/w)	2 flavored e-liquids	ISO 20768:2018 (Dur: 3 sec; Int: 30 sec; Vol: 55 mL; Puffs: 50; Sets: 1)	LC-MS/MS	S
Poynton et al., 2017 (28)	UK	Yes (Services were provided by BAT)	EC01: Closed-modular system	EC01 Brand: Vype, ePen; consisted of rechargeable battery and replaceable cartomizer with e-liquid tank and atomizer (Volt: 3.6V; NiCr Alloy; USB-Rechargeable battery 650 mAh)	EC01: Flavor: Blended Tobacco	1 e-liquid flavor variant	CORESTA (Dur: 3 sec; Int: 30 sec; Vol: 55 mL; Puffs: 100; Sets: 2 of 100 puffs each)	LC-MS/MS	G
Rudd et al., 2020 (29)	UK	Yes (Imperial Brands)	EC01: Pod	EC01 Brand: Myblu; closed pod-system consisting of a rechargeable battery and a replaceable e-liquid containing pod (Res: 1.3Ω; Rechargeable battery 350mAh)	EC01: Nic: 1.6%; Flavor: Tobacco	1 flavor	CORESTA No.81 (Dur: 3 sec; Int: 30 sec; Vol: 55 mL; Puffs: 150; Sets: 3 of 50 each)	LC-MS/MS	S
Tayyarah et al., 2014 (30)	US	Yes (All authors were employed by Lorillard Tobacco Company)	EC01: Disposable EC02 & EC03: Rechargeable	EC01 Brand: blu; ECs were manufactured by blu eCigs EC02 Brand: blu; ECs were manufactured by blu eCigs EC03 Brand: SKYCIG; ECs were manufactured by SKYCIG	EC01: Nic: 24mg/unit; Flavors: Classic Tobacco (7%) and Magnificent Menthol (5%); Glycerin: blu CTD: 75%; blu MMD: 82% EC02: Nic: 16mg/unit; Flavor: Cherry Crush (7%); Glycerin: 77% EC03: Nic: 18mg/unit; Flavors: Classic Tobacco Bold (1%) and Crown Menthol Bold (4%); PG/VG: SKYCIG CTB: 67%/24%; SKYCIG CMB: 66%/21%	NR	Health Canada Method T-115 (Dur: NR; Int: 2/min; Vol: 55 mL; Puffs: 99; Sets: 1)	LC-MS/MS	S

^*^Risk of Bias assessed through QualySyst grading tool: S = Strong Score Summary of > 0.80; G, Good Score Summary of 0.71-0.79.

Abbreviations: Ω, Ohm; BAT, British American Tobacco Investments Ltd.; cm^3^, Cubic centimeter; CORESTA, Cooperation Centre for Scientific Research Relative to Tobacco; Cr, Chromium; Dur, Puff duration; EC, Electronic Cigarette; GC, Gas Chromatography; Int, Intervals between puffs; ISO, International Organization for Standardization; LC, Liquid Chromatography; mAh, Milliampere-hour; MEIN, Ministry of Science and Higher Education of Poland; mg, Milligram(s); mL, Milliliter(s); MS/MS, Tandem Mass Spectrometry; ND, Not Detected; Ni, Nickel; Nic: Nicotine Content; NiCr, Nichrome; NiFe: Nickel-Iron; NIH, National Institutes of Health; NR, Not Reported; PG, Propylene glycol; Puffs, Number of puffs over total sets; Res, Resistance; sec, Second(s); Sets, Number of puffing sets collected; UK, United Kingdom; US, United States; USB, Universal Serial Bus; Vol, Puff volume; V, Volt; Volt, Voltage; VG, Vegetable Glycerin; W, Watt; Watt, Wattage.

Of the 13 included studies, three studies were published in 2020 (23, 26, 29), two in 2022 (16, 27), 2021 (24, 31), and 2014 (14, 30), and one each in 2024 (22), 2018 (32), 2017 (28), and 2016 (25). The majority of the included studies (53.8%) were carried out in the UK (23–29), five studies were conducted in the US (16, 22, 30–32), and one study was conducted in Poland and the US (14). Eleven of the included studies were funded by the tobacco industry, with sponsors including British American Tobacco (BAT) (23–28), Imperial Brands (29), JUUL Labs, Inc (22, 31)., Altria Client Services LLC (16), and Lorillard Tobacco Company (30).

The studies investigated a range of e-cigarette device types, including closed pod-based systems (e.g. JUUL, Vype ePod, Vype ePen, and Myblu™), disposables (e.g. blu), rechargeables (e.g. blu, SKYCIG), and cig-a-likes (e.g. MarkTen, MarkTenXL, Joye 510, DSE 910, Trendy 808, Nicore M401, Mild 201, Colinss Age, Premium PR111, Ecis 510, and Intellicig Evolution). One study described the e-cigarette as a product that uses a stainless-steel mesh distiller plate with a microporous structure to heat and aerosolize e-liquid, optimizing wicking and preventing overheating, without specifying its brand or type (26).

Nicotine concentrations in e-liquids of the tested products ranged from 5 mg/mL (26) to 57 mg/mL (27), with formulations spanning tobacco, menthol/mint, and fruit flavors. PG and VG content in the e-liquids varied widely among studies, with PG content ranging from 24% (24) to 67% (30) and VG ranging from 31% (23) to 82% (30). All studies compared e-cigarette aerosol emissions against reference cigarettes (e.g., Kentucky 1R6F/3R4F, B&H Skyblu, Marlboro, Lambert & Butler). Puffing regimes were predominantly based on standardized protocols such as CORESTA Method No. 81 (ISO 20768), with puff durations ranging from 3 to 6 seconds and puff volumes between 55 and 110 mL (16, 22–29, 31). Two studies followed Health Canada Test Method, in which puffs were taken twice per minute in a volume of 55 mL (30, 32). Another study’s puffing regimen was based on results of inhalation topography measurement among ten regular e-cigarette users, whereby the puff duration was 1.8 sec, puff interval was 10 sec, and puff volume was 70 mL (14).

Across the 13 studies, TSNAs in e-cigarette aerosols and cigarette smoke were quantified using analytical methods such as liquid chromatography-tandem mass spectrometry (LC-MS/MS) (23–32), ultra-performance liquid chromatography–mass spectrometry (UPLC-MS) (14, 16), and gas chromatography-mass spectrometry (GC-MS/MS) (22).

### 3.2 NNK and NNN levels in e-cigarette aerosols

#### 3.2.1 Studies with levels below LOD/LOQ

Across majority of studies, levels of NNK and NNN in e-cigarette aerosol were consistently reported as being below the LODs, in contrast to the substantial yields of these nitrosamines measured in reference cigarette smoke.

Four studies assessed emissions from closed-system Vype devices, including Vype ePen2 (23, 24), ePen3 (23), ePod 1.0 (27), and ePen I (28). Cunningham et al. (23) found that NNK and NNN were below detection limits across all Vype ePen2 and ePen3 variants, regardless of benzoic acid level in e-liquids, while reference cigarettes produced measurable TSNA levels. Similar findings were reported by Margham et al. (24) for Vype ePen2 across multiple flavors, including Golden Tobacco, Dark Cherry, and Crisp Mint. Pinto et al. (27) investigated a fourth generation Vype ePod 1.0 with Berry Blast e-liquid (18 and 57 mg/mL nicotine), confirming negligible TSNA formation in newly adopted pod devices with innovative ceramic wick-based technology. Poynton et al. (28) assessed the Vype ePen I and reported similar findings. Across all four studies, NNK and NNN emissions from Vype products were reduced by ~99.9% per puff relative to conventional cigarette smoke.

Another closed-pod system study by Rudd et al. (29) further showed that NNK and NNN emitted from Myblu™ e-cigarettes were below limits of detection, unlike the 3R4F reference cigarette which produced significant TSNA levels. On a per-puff basis, nitrosamine levels in e-cigarette aerosol were reduced by >99% compared with those in cigarette smoke. Additionally, a rechargeable e-cigarette consisting of a disposable cartridge studied by Nicol et al. (26) emitted NNK and NNN at levels below detection limits whereas 3R4F reference cigarettes exhibited measurable levels. In the study by Chen et al. (31), analyses of 53 aerosol constituents from four JUUL products currently on the US market were compared to historical results from 3R4F using non-intense and intense puffing regimens. Across the four JUUL products and two puffing regimes, NNK and NNN were below detection limits compared to measurable levels in cigarettes and were around 99% lower than cigarettes. Similarly, Tayyarah et al. (30) examined disposable and rechargeable blu eCigs as well as rechargeable SKYCIG e-cigarettes, and across all tested flavors and device types, NNK and NNN were below detection limits while levels were measurable in cigarettes.

#### 3.2.2 Studies with trace levels

Only five investigations reported quantifiable concentrations of NNK and/or NNN in certain e-cigarette emissions.

Goniewicz et al. (14) examined a range of cartridge and cartomizer-based devices available in Poland and the UK. These devices were first generation cig-a-like products that are today considered outdated (21, 33). NNK and NNN were detected at trace levels in select devices, with NNN levels ranging from 0.8 ng to 4.3 ng and NNK from 1.1 ng to 28.3 ng per one e-cigarette (150 puffs). NNN and NNK levels found in the smoke from a conventional cigarette were 380-fold and 40-fold higher than levels in the vapor of an e-cigarette, respectively.

Jin et al. (16) studied eight cig-a-like devices available in the US market, including MarkTenXL e-cigarettes in Classic and Menthol flavors. NNK and NNN were either below detection limits or present at trace levels, with quantified levels ranging from 0.0015 to 0.16 ng/puff for NNK and 0.003 to 0.15 ng/puff for NNN. Except for NNK in one product (22.5 ng/e-cigarette), all TSNA levels in e-cigarette aerosols were lower than those reported in the smoke of 50 US commercial cigarettes (NNK 13-122.4 ng/cigarette; NNN 18.6-171.1 ng/cigarette) obtained from the literature. In addition, the study demonstrated that fortifying e-liquids with nitrite increased TSNA formation, with levels correlating positively with the added nitrite concentration. The authors suggested a potential role of impurities in TSNA generation as higher NNK and NNN levels were associated with devices whose e-liquids contained nitrite and minor alkaloid impurities.

Leigh et al. (32) examined TSNAs between MarkTen e-cigarettes, heated tobacco products, and cigarettes. Although the data were not explicitly presented in the text or in tables, an estimation based on the figure presented revealed that NNK was not detectable in e-cigarettes (versus cigarettes measured at ~6.604 ng/puff). On the other hand, NNN for e-cigarettes was measured to be around 0.017 ng/puff, while cigarettes had a substantially higher level of NNN (~14.2 ng/puff).

In the study conducted by Cook et al. (22), emissions were tested from a range of JUUL2 e-liquid flavors. While neither NNK nor NNN were detected in most of the tested flavors when evaluated under both non-intense and intense puffing regimens, an exception was observed with the Ruby Menthol flavor. Measurable NNN concentrations were detected at an average of 0.01 ± 0.0005 µg under both non-intense (130 puffs) and intense (80 puffs) puffing conditions. Furthermore, the same e-liquid flavor yielded NNN levels of 0.004 ± 0.0003 µg per 76 puffs after three months of storage when evaluated under the intense regimen. The non-intense regimen followed ISO 20768:2018 specifications (3-second second puff duration, 30-second interpuff interval, 55-mL puff volume), while the intense regimen also complied with ISO 20768:2018 but with extended puff duration (6 seconds) and larger puff volume (110 mL). The authors concluded that JUUL2 e-cigarettes emitted overall TSNA levels that were markedly reduced by more than 99% compared with cigarette smoke under both non-intense and intense puffing regimens.

Similarly, Margham et al. (25) reported quantifiable nitrosamine levels from the Vype ePen, closed-system e-cigarette operated at 3.6 V. NNK concentrations in e-cigarette aerosols (0.01 ng/puff) were comparable to those detected in the air blank sample (0.016 ng/puff) but substantially lower than those from the 3R4F Kentucky reference cigarette (26.67 ng/puff). For NNN, the Vype ePen emitted 0.054 ng/puff compared to 0.014 ng/puff in the air blank sample and 24.97 ng/puff in the reference cigarette smoke. The authors indicated that the per-puff nitrosamine emissions from the e-cigarette were 99.8% lower than those from the 3R4F reference cigarette. They further suggested that the trace levels of NNN detected in the e-cigarette aerosol likely originated from impurities present in the Pharmacopoeia-grade nicotine used in the formulation.

### 3.3 Quality assessment

Nine studies (69.2%) were rated as “strong” in overall research quality using the QualSyst tool (22–27, 29–31), whereas three studies (23.1%) had a “good” rating (14, 16, 28), and one study (7.7%) had an “adequate” rating (32). Among the studies rated as “strong”, one study had a score of 0.96 (27), two had scores of 0.93 (25, 26), two had scores of 0.89 (23, 24), two had scores of 0.86 (22, 30), and two had a score of 0.82 (29, 31). Of the studies with “good” rating, one study had a score of 0.75 (14), and two had scores of 0.71 (16, 28). The study with an “adequate” rating, had a score of 0.64 (32). **Supplementary Material** (**Table 2**) provides the individual item scores for each included study.

Collectively, studies had insufficient reporting on key aspects of design, methodology, analysis, and reporting. Areas of incomplete reporting included device characteristics (11 studies; 14, 16, 22, 23, 25, 26, 28-32), e-liquid characteristics (7 studies; 14, 16, 22, 28, 29), overall sample descriptions (4 studies; 14, 16, 30, 32), appropriate assessment of background correction (4 studies; 16, 22, 29, 32), quality control measures (12 studies; 14, 16, 22-30, 32), limitations in puffing regimens (2 studies; 14, 30), aerosol collection and analytical methods (2 studies; 24, 28), some estimate of variance for certain results (3 studies; 16, 31, 32), LOD or LOQ levels (4 studies; 14, 16, 28), and measures of central tendency for some results (2 studies; 16, 32).

The variation in scores across the 13 studies emphasize the need for standardized reporting of device and e-liquid characteristics, vaping conditions, analytical procedures, and quality control measures in studies evaluating e-cigarette emissions.

## 4 Discussion

This analysis sought to clarify the extent of user exposure to TSNAs, particularly NNK and NNN, in e-cigarette emissions compared to combustible cigarette smoke. Across the 13 studies reviewed, validated analytical methods demonstrated that levels of TSNAs in e-cigarette aerosols were consistently several orders of magnitude lower than those measured in conventional cigarette smoke. Collectively, these findings delineate a clear toxicological gradient, with combustible cigarettes producing the highest exposures, e-cigarettes producing substantially lower exposures that fall below detection or near background air levels in most modern devices, and only a minority of older products yielding trace but measurable amounts.

The mechanistic evidence helps explain this pattern. Unlike in combustible cigarettes, where curing and pyrolysis generate elevated levels of TSNAs, e-cigarettes do not produce these compounds during use. Instead, their presence reflects impurities introduced during nicotine extraction or the nitrosation of minor alkaloids in the presence of nitrite. Jin and colleagues (16) demonstrated that in early cig-a-like products, the appearance of NNK and NNN was entirely attributable to nitrite contamination. Adding nitrite to nicotine solutions generated measurable TSNAs over time, which increased further with heat; nitrite-free formulations yielded either undetectable or background-level values in both liquid and aerosol phases. Independent investigations reinforce this interpretation. Farsalinos and colleagues (12) systematically compared TSNA concentrations in e-liquids and the aerosols generated from them. They found that aerosol levels never exceeded those already present in the e-liquid, and in several cases no TSNAs were detected in the aerosol at all despite trace levels in the e-liquid. This indicates that the process of vaporization does not create additional nitrosamines; rather, what appears in the aerosol reflects direct transfer of pre-existing contaminants from the liquid. Thus, when TSNAs are detected in e-cigarette emissions, their source is almost certainly the quality of the liquid ingredients, not the act of vaping itself. Converging evidence comes from product-surveillance studies conducted without cigarette comparators. In a multi-brand evaluation performed under CORESTA-consistent conditions, TSNAs in e-liquids were generally below quantification limits and remained below quantification in the corresponding aerosols; the investigators also emphasized the necessity of air blanks to prevent attributing background contamination to the device signal (34). A separate analytical validation program reported TSNAs in e-cigarette aerosols at levels observed in pharmaceutical-grade nicotine and provided transparent limits of quantitation and detection for NNK and NNN, enabling clearer interpretation of non-detects (13).

The clinical evidence aligns with these chemical findings. Biomarker studies consistently show that when smokers switch completely to e-cigarettes, exposure to TSNAs is markedly reduced. Shahab and colleagues (35) reported that urinary NNAL (4-(methylnitrosamino)-1-(3-pyridyl)-1-butanol) concentrations in long-term exclusive e-cigarette users were almost indistinguishable from those in users of licensed nicotine-replacement therapy, and far below the levels observed in smokers. Goniewicz and colleagues (36) observed similar patterns in large US cohorts, demonstrating that reductions in nitrosamine biomarkers are substantial when cigarette use is replaced entirely by vaping.

The evidence presented in this secondary analysis comparing e-cigarettes and cigarettes indicates that TSNA exposure from e-cigarettes is not an inherent property of vaping but largely reflects the quality of the e-liquid. Meaningful reductions in internal exposure to carcinogens are seen when combustible tobacco is replaced entirely with e-cigarettes. Communicating this nuance, that risk is lower but not absent and that benefit depends on both reputable products and full switching, provides a balanced and evidence-based framework for guiding decisions about use.

One possible reason the perception of TSNA exposure from e-cigarettes continues to circulate is the reliance on observational cohort studies. These analyses are valuable but have important limitations. Most e-cigarette users in such cohorts are former smokers, biomarker half-lives vary widely, and patterns of use are often self-reported in ways that make it difficult to distinguish exclusive from dual use. Under these conditions, residual effects of past smoking or concurrent cigarette use may be misattributed to e-cigarettes, creating the impression that vaping itself is a significant source of nitrosamines.

Our analysis helps explain why this interpretation may not be reliable. Across the studies we examined, TSNAs were largely absent from aerosols, with only trace amounts detected under limited conditions. Observational cohorts seldom capture information on the e-liquid or product being used, leaving out the factor that appears most decisive. This disconnect between what is measured in large population studies and what determines exposure helps to sustain the perception that e-cigarettes are substantial sources of TSNAs. When observational evidence is set alongside experimental and mechanistic findings, the picture becomes clearer. Nitrosamine exposure is driven by e-liquid and manufacturing quality and by the continued use of combustible cigarettes, not by vaping in isolation.

Most included studies were rated as strong in methodological quality, though several limitations warrant consideration. Most investigations were funded by the tobacco industry, which can be regarded as a source of concern. However, within this evidence base, methodological standards were generally high, with extensive use of validated analytical methods that reduce the likelihood of systematic bias. The two studies included in this analysis that were not industry funded both found e-cigarette emissions to contain only trace amount of NNK and NNN, which was substantially lower than cigarettes (14, 32). These results therefore concur with conclusions made by industry-funded studies. Nonetheless, independent replication remains important, but the overall rigor of the included studies lowers concern that funding source compromised the findings. Remaining limitations relate primarily to differences in reporting of device specifications, puffing regimens, and limits of detection, all of which hinder cross-study comparison. Although standardized methods such as CORESTA 81 (ISO 20768) were frequently applied, variation in puff volume, duration, and replicates complicates direct synthesis. The lack of uniform reporting of LOD and LOQ further complicates interpretation, particularly of “non-detect” findings. Greater transparency and standardization would strengthen the evidence base and allow for more reliable comparative analysis.

This study also carries both strengths and limitations. A key strength is the comprehensive identification of relevant studies, which was possible given the systematic approach used by the systematic review of e-cigarette emissions. By identifying and synthesizing data on NNK and NNN, this analysis addresses a gap in the cancer literature where direct comparisons with cigarettes have not previously been collated. The use of explicit eligibility criteria and structured quality assessment further enhances internal validity. At the same time, the analysis is constrained by the small number of eligible studies, by reliance on published data rather than raw laboratory outputs, and by heterogeneity in devices, e-liquids, and reporting formats including the reporting of LOD and LOQ levels. These constraints limited the ability to conduct a meta-analysis and necessitated a descriptive synthesis. Furthermore, given the rapidly evolving advances in e-cigarette devices, another analysis on this topic in the near future may reveal additional insights. Taken together, these limitations indicate that the results should be interpreted as indicative of consistent patterns across studies, rather than precise quantitative estimates of exposure.

While the short-term toxicological evidence is compelling, important gaps remain. Longitudinal epidemiological studies linking e-cigarette use to cancer outcomes will be essential, but these require extended follow-up with improved methods for reporting and measuring of respondent’s tobacco use histories, outcomes, and the exact device and e-liquid used. In the interim, progress can be made by standardizing TSNA measurement and reporting across laboratories, expanding emission studies to include a broader range of products and formulations, and investigating how product storage or aging may influence nitrosamine formation. Independent replication of existing findings will further build confidence and reduce reliance on industry-sponsored data.

Taken together, this analysis highlights that TSNA exposure from e-cigarettes is not an inevitable property of vaping but depends on the chemical quality of the e-liquid. Clearer reporting, more independent research, and sustained attention to methodological rigor will help move the field beyond the limitations of observational cohorts and toward a firmer understanding of the true cancer-related risks of e-cigarettes.

## 5 Conclusions

In conclusion, this analysis demonstrates that NNK and NNN levels in e-cigarette aerosols are consistently and substantially lower than those found in combustible cigarette smoke, with reductions typically exceeding 99%. In many modern devices, nitrosamines are undetectable, and where detected, they appear at trace levels likely reflecting nicotine purification and ingredient quality rather than inherent properties of vaping. For oncology practice, the evidence indicates that exclusive e-cigarette use confers a profound reduction in exposure to two of the most important tobacco-specific carcinogens.

Nevertheless, reductions are not synonymous with elimination. The variability introduced by dual use, unregulated products, and incomplete ingredient purification underscores the need for careful clinical guidance and regulatory oversight. The role of the oncology community is to articulate this evidence with clarity, precision, and balance, recognizing both the substantial benefits of reduced exposure and the residual uncertainties that only long-term studies will resolve.

## Data Availability

The original contributions presented in the study are included in the article/**supplementary material**. Further inquiries can be directed to the corresponding author.
