# Supplementary material for: Tobacco-specific nitrosamine exposure from electronic cigarettes versus combustible cigarettes: an ad hoc analysis within a systematic review of emission studies
Source: Front Oncol. 2025 Dec 12;15:1729107. doi: 10.3389/fonc.2025.1729107 (PMC12740905; doi:10.3389/fonc.2025.1729107)
Supplement: Supplementary file 1 [file Table1.docx]

## **Table 1. Characteristics of Included Studies and Outcome Measures (n=13)**

| Study Characteristics | EC Device Characteristics | Experimental Conditions | Outcomes |
| --- | --- | --- | --- |
| Chen et al. (2021)  Country  US  Laboratory site  Canada  Source of study funding  No external funding; all authors employed by Juul Labs, Inc. | EC01  Device type: Pod  Device brand and model: JUUL Pods  Device purchase date: NR  Additional type/brand notes: EC was tested within 2 months of pod filling.  Voltage: NR  Heating element material: NR  Heating element temperature: NR  Resistance: NR  Power/wattage: NR  Battery type: NR  Flavor: Virginia Tobacco and Menthol  Nicotine content: 3% and 5% (35 and 59 mg/mL)  PG/VG content: NR  Additional e-liquid notes: N/A | Number of devices  N=1  Number of samples  N=30 (10 replicates of 3 lots of each JUUL flavor/concentration for each puffing regimen)  Number of replicates  N=10 replicate measurements  Control/comparator  3R4F Kentucky Research Cigarette  Blank  Puffing machine  Standardized linear puffing machine  Puffing sets  N=2 (Non-intense and Intense); each had three 50-puff collections  Puffing regimen  Method: Non-intense ISO 20768  Puff duration: 3 sec  Intervals between puffs: 30 sec  Puff volume: 55 mL  Number of puffs: 50  Puff profile: NR  Air flow speed: NR  Method: Intense regimen  Puff duration: 6 sec  Intervals between puffs: 30 sec  Puff volume: 110 mL  Number of puffs: 50  Puff profile: NR  Air flow speed: NR  Aerosol Collection Method  Aerosols were collected on pads that were spiked with internal standard solution containing 4 TSNA analogues and were extracted into ammonium acetate solution.  Analytical method(s)  LC-MS/MS (ESI) | **Levels of nitrosamines, Mean**  **NNK (mg/puff):**  *Non-intense puffing regimen*  JUUL (Virginia Tobacco, 3%): ≤7.53×10^-09^  JUUL (Virginia Tobacco, 5%): ≤7.53×10^-09^  JUUL (Menthol, 3%): ≤7.53×10^-09^  JUUL (Menthol, 5%): ≤7.53×10^-09^  Cigarette*: 1.13×10^-04^ mg/cig  Blank: NR  LOD: 0.02 ng  LOQ: 0.05 ng  *Intense puffing regimen*  JUUL (Virginia Tobacco, 3%): ≤7.53×10^-09^  JUUL (Virginia Tobacco, 5%): ≤7.53×10^-09^  JUUL (Menthol, 3%): ≤7.53×10^-09^  JUUL (Menthol, 5%): ≤7.53×10^-09^  Cigarette*: 2.95×10^-04^ mg/cig  Blank: NR  LOD: 0.02 ng  LOQ: 0.05 ng  **NNN (mg/puff):**  *Non-intense puffing regimen*  JUUL (Virginia Tobacco, 3%): ≤4.92×10^-09^  JUUL (Virginia Tobacco, 5%): ≤4.92×10^-09^  JUUL (Menthol, 3%): ≤4.92×10^-09^  JUUL (Menthol, 5%): ≤4.92×10^-09^  Cigarette*: 1.31×10^-04^ mg/cig  Blank: NR  LOD: 0.02 ng  LOQ: 0.05 ng  *Intense puffing regimen*  JUUL (Virginia Tobacco, 3%): ≤4.92×10^-09^  JUUL (Virginia Tobacco, 5%): ≤4.92×10^-09^  JUUL (Menthol, 3%): ≤4.92×10^-09^  JUUL (Menthol, 5%): ≤4.92×10^-09^  Cigarette*: 3.37×10^-04^ mg/cig  Blank: NR  LOD: 0.02 ng  LOQ: 0.05 ng  ** Values for mainstream smoke constituents were taken from Jaccard et al. 2019.* |
| Cook et al. (2024)  Country  US  Laboratory site  US  Source of study funding  No external funding; all authors employed by Juul Labs. Testing at Enthalpy Analytical and Charles River was paid in full by Juul Labs, Inc. | EC01  Device type: Pod  Device brand and model: JUUL2 System  Device purchase date: NR  Additional type/brand notes: Temperature-regulated consisting of a closed (1.2 mL capacity) pod and technologically advanced device (i.e., smartphone-compatible with device locking, tracking, and usage analytic features); Launched in the UK in September 2021.  Voltage: NR  Heating element material: NR  Heating element temperature: NR  Resistance: NR  Power/wattage: NR  Battery type: NR  Flavor: Virginia Tobacco, Crisp Menthol, Polar Menthol, Autumn Tobacco, Ruby Menthol, and  Summer Menthol  Nicotine content: 18 mg/mL  PG/VG content: NR  Additional e-liquid notes: N/A | Number of devices  N=1*  Number of samples  N=6 formulations  Number of replicates  N=5 per analytical assay per  time point  Control/comparator  1R6F Reference Cigarette  Blank  Puffing machine  Linear 20-port EC puffing machine (Cerulean SM450e, Milton Keynes)  Puffing sets  N=2 (Intense and Non-intense) at T0 and T3^†^  Puffing regimen  Method: ISO 20768:2018 (non-intense)  Puff duration: 3 sec  Intervals between puffs: 30 sec  Puff volume: 55 mL  Number of puffs: 100-150  Puff profile: Square wave  Air flow speed: NR  Method: ISO 20768:2018 (intense)  Puff duration: 6 sec  Intervals between puffs: 30 sec  Puff volume: 110 mL  Number of puffs: 60-90  Puff profile: Square wave  Air flow speed: NR  Aerosol Collection Method  Aerosols were collected by passing aerosol through a 55 mm GFFP and extracted with deionized water.  Analytical method(s)  GC-MS/MS  **One commercially representative pre-production lot per formulation was tested.*  *^†^T0 = Baseline; T3 = After 3 months of storage* | **Levels of nitrosamines, Mean±SD**  **NNK (µg/collection):**  *EC01*  *At T0 – Non-Intense Regimen:*  Virginia Tobacco (150 puffs): <LOQ  LOD: 0.003  LOQ: 0.03  Crisp Menthol (125 puffs): <LOQ  LOD: 0.003  LOQ: 0.03  Polar Menthol (100 puffs): <LOQ  LOD: 0.003  LOQ: 0.03  Autumn Tobacco (128 puffs): <LOQ  LOD: 0.001  LOQ: 0.003  Ruby Menthol (130 puffs): <LOQ  LOD: 0.001  LOQ: 0.003  Summer Menthol (126 puffs): <LOQ  LOD: 0.001  LOQ: 0.003  *At T0 – Intense Regimen:*  Virginia Tobacco (86 puffs): <LOQ  LOD: 0.002  LOQ: 0.03  Crisp Menthol (81 puffs): <LOQ  LOD: 0.00  LOQ: 0.03  Polar Menthol (81 puffs): <LOQ  LOD: 0.002  LOQ: 0.03  Autumn Tobacco (80 puffs): <LOQ  LOD: 0.001  LOQ: 0.003  Ruby Menthol (80 puffs): <LOQ  LOD: 0.001  LOQ: 0.003  Summer Menthol (86 puffs): <LOQ  LOD: 0.001  LOQ: 0.003  *At T3 – Non-Intense Regimen*:*  Autumn Tobacco (128 puffs): <LOQ  LOD: 0.001  LOQ: 0.003  Ruby Menthol (127 puffs): <LOQ  LOD: 0.001  LOQ: 0.003  Summer Menthol (127 puffs): <LOQ  LOD: 0.001  LOQ: 0.003  *At T3 – Intense Regimen*:*  Autumn Tobacco (73 puffs): <LOQ  LOD: 0.001  LOQ: 0.003  Ruby Menthol (76 puffs): <LOQ  LOD: 0.001  LOQ: 0.003  Summer Menthol (80 puffs): <LOQ  LOD: 0.001  LOQ: 0.003  *Cigarette (ng/cigarette)*  Non-Intense (ISO 3308:2012): 71  Intense (ISO 20778:2018): 187  Blank: NR  **NNN (µg/collection):**  *EC01*  *At T0 – Non-Intense Regimen:*  Virginia Tobacco (150 puffs): <LOQ  LOD: 0.003  LOQ: 0.03  Crisp Menthol (125 puffs): <LOQ  LOD: 0.003  LOQ: 0.03  Polar Menthol (100 puffs): <LOQ  LOD: 0.003  LOQ: 0.03  Autumn Tobacco (128 puffs): <LOQ  LOD: 0.001  LOQ: 0.003  Ruby Menthol (130 puffs): 0.01±0.0005  LOD: 0.001  LOQ: 0.003  Summer Menthol (126 puffs): <LOQ  LOD: 0.001  LOQ: 0.003  *At T0 – Intense Regimen:*  Virginia Tobacco (86 puffs): <LOQ  LOD: 0.003  LOQ: 0.03  Crisp Menthol (81 puffs): <LOQ  LOD: 0.00  LOQ: 0.03  Polar Menthol (81 puffs): <LOQ  LOD: 0.003  LOQ: 0.03  Autumn Tobacco (80 puffs): <LOQ  LOD: 0.001  LOQ: 0.003  Ruby Menthol (80 puffs): 0.01±0.0005  LOD: 0.001  LOQ: 0.003  Summer Menthol (86 puffs): <LOQ  LOD: 0.001  LOQ: 0.003  *At T3 – Non-Intense Regimen*:*  Autumn Tobacco (128 puffs): <LOQ  LOD: 0.001  LOQ: 0.003  Ruby Menthol (127 puffs): <LOQ  LOD: 0.001  LOQ: 0.003  Summer Menthol (127 puffs): <LOQ  LOD: 0.001  LOQ: 0.003  *At T3 –Intense Regimen*:*  Autumn Tobacco (73 puffs): <LOQ  LOD: 0.001  LOQ: 0.003  Ruby Menthol (76 puffs): 0.004±0.0003  LOD: 0.001  LOQ: 0.003  Summer Menthol (80 puffs): <LOQ  LOD: 0.001  LOQ: 0.003  *Cigarette (ng/cigarette)*  Non-Intense (ISO 3308:2012): 85  Intense (ISO 20778:2018): 212  Blank: NR  **Only Autumn Tobacco, Ruby Menthol, and Summer Menthol were tested at T3.* |
| Cunningham et al. (2020)  Country  UK  Laboratory site  UK  Source of study funding  BAT | EC01  Device type: NR  Device brand and model: Vype ePen2  Device purchase date: NR  Additional type/brand notes:  The EC consists of a reusable section containing a rechargeable battery and an actuation button, a disposable flavor cartridge, and a mouthpiece cover with a silica rope wick.  Voltage: NR  Heating element material: NiCr  Heating element temperature: NR  Resistance: NR  Power/wattage: 2.8 W and 4.4 W  Battery type: Rechargeable; 650 mAh  Flavor: Blended Tobacco  Nicotine content: 18 mg/mL (1.78% w/w)  PG/VG content: 25.00%/48.22% (w/w)  Additional e-liquid notes: N/A  EC02  Device type: Closed system  Device brand and model: Vype ePen3  Device purchase date: NR  Additional type/brand notes:  The battery electronics has a protect circuit board to protect against short-circuiting, low or high charging voltage, over current, and over charging. It stops battery power to the coil after 8 sec, thereby limiting dry-puff events.  Voltage: NR  Heating element material: NiFe alloy (cotton wick)  Heating element temperature: NR  Resistance: 1.95-2.36 Ω  Power/wattage: 5.9 W  Battery type: Rechargeable; 650 mAh  Flavor:  ePen3 18: Blended Tobacco  ePen3 12 Low BA, ePen3 18 Medium BA, and ePen3 30 High BA: MasterBlend  Nicotine content: 12, 18, and 30 mg/mL  PG/VG content:  ePen3 BT 18: 54.00%/34.22% (w/w)  ePen3 MB 12 Low BA: 54.25%/34.57% (w/w)  ePen3 MB 18 Medium BA: 54.73%/33.5% (w/w)  ePen3 MB 30 High BA: 56.06%/31.2% (w/w)  Additional e-liquid notes: N/A | Number of devices  N=2  Number of samples  N=5 EC variants  Number of replicates  N=5 per observation for all analyses  Control/comparator  1R6F Kentucky Reference Cigarette  B&H Skyblue cigarette  Blank  Puffing machine  Linear smoking machine  Puffing sets  N=1  Puffing regimen  Method: CORESTA Method No.81 (ISO 20768)  Puff duration: 3 sec  Intervals between puffs: 30 sec  Puff volume: 55 mL  Number of puffs: 50  Puff profile: Square wave  Air flow speed: NR  Aerosol Collection Method  Aerosol was trapped on a 44-mm GFFP followed by a cryogenic (≤ 35°C) trap (impinger) containing 20 mL acetonitrile.  Analytical method(s)  LC-MS | **Levels of nitrosamines, Mean±SD**  **NNK (pg/puff):**  ePen2 BT 18*: BDL  ePen3 BT 18: BDL  ePen3 MB 12 Low BA: BDL  ePen3 MB 18 Medium BA: BDL  ePen3 MB 30 High BA: BDL  LOD: 15.05  LOQ: 50.18  Ky1R6F cigarette^†^: 20.97±1.075 ng/puff  LOD: 0.0270 ng/puff  LOQ: 0.0899 ng/puff  B&H Skyblue cigarette: 9.093±2.023 ng/puff  LOD: 0.0292 ng/puff  LOQ: 0.0973 ng/puff  Blank: NQ  **NNN (pg/puff):**  ePen2 BT 18*: BDL  ePen3 BT 18: BDL  ePen3 MB 12 Low BA: BDL  ePen3 MB 18 Medium BA: BDL  ePen3 MB 30 High BA: BDL  LOD: 9.85  LOQ: 32.82  Ky1R6F cigarette^†^: 22.69±1.398 ng/puff  LOD: 0.0176 ng/puff  LOQ: 0.0588 ng/puff  B&H Skyblue cigarette: 9.105±2.116 ng/puff  LOD: 0.0191 ng/puff  LOQ: 0.0636 ng/puff  Blank: NQ  ** The EC was operated at the 4.4 W setting.*  *† Data extracted from Jaccard et al (2019).* |
| Goniewicz et al. (2014)  Country  Poland & US  Laboratory site  Poland & US  Source of study funding  MEIN and NIH | EC01  Device type: NR  Device brand and model: Joye 510  Device purchase date: NR  Additional type/brand notes: The EC was purchased online from Poland.  Voltage: NR  Heating element material: NR  Heating element temperature: NR  Resistance: NR  Power/wattage: NR  Battery type: NR  Flavor: Marlboro  Nicotine content: 4 mg/mL  PG/VG content: NR  Additional e-liquid notes: N/A  EC02  Device type: NR  Device brand and model: Janty eGo  Device purchase date: NR  Additional type/brand notes: The EC was purchased online from Poland.  Voltage: NR  Heating element material: NR  Heating element temperature: NR  Resistance: NR  Power/wattage: NR  Battery type: NR  Flavor: Marlboro  Nicotine content: 16 mg/mL  PG/VG content: NR  Additional e-liquid notes: N/A  EC03  Device type: NR  Device brand and model: Janty Dura  Device purchase date: NR  Additional type/brand notes: The EC was purchased online from Poland.  Voltage: NR  Heating element material: NR  Heating element temperature: NR  Resistance: NR  Power/wattage: NR  Battery type: NR  Flavor: Marlboro  Nicotine content: 16 mg/mL  PG/VG content: NR  Additional e-liquid notes: N/A  EC04  Device type: NR  Device brand and model: DSE 910  Device purchase date: NR  Additional type/brand notes: The EC was purchased online from Poland.  Voltage: NR  Heating element material: NR  Heating element temperature: NR  Resistance: NR  Power/wattage: NR  Battery type: NR  Flavor: Regular  Nicotine content: 16 mg/mL  PG/VG content: NR  Additional e-liquid notes: N/A  EC05  Device type: NR  Device brand and model: Trendy 808  Device purchase date: NR  Additional type/brand notes: The EC was purchased online from Poland.  Voltage: NR  Heating element material: NR  Heating element temperature: NR  Resistance: NR  Power/wattage: NR  Battery type: NR  Flavor: Trendy  Nicotine content: 18 mg/ml  PG/VG content: NR  Additional e-liquid notes: N/A  EC06  Device type: NR  Device brand and model: Nicore M401  Device purchase date: NR  Additional type/brand notes: The EC was purchased online from Poland.  Voltage: NR  Heating element material: NR  Heating element temperature: NR  Resistance: NR  Power/wattage: NR  Battery type: NR  Flavor: Marlboro  Nicotine content: 18 mg/mL  PG/VG content: NR  Additional e-liquid notes: N/A  EC07  Device type: NR  Device brand and model: Mild 201  Device purchase date: NR  Additional type/brand notes: The EC was purchased online from Poland.  Voltage: NR  Heating element material: NR  Heating element temperature: NR  Resistance: NR  Power/wattage: NR  Battery type: NR  Flavor: Marlboro  Nicotine content: 18 mg/mL  PG/VG content: NR  Additional e-liquid notes: N/A  EC08  Device type: NR  Device brand and model: Colinss Age  Device purchase date: NR  Additional type/brand notes: The EC was purchased online from Poland.  Voltage: NR  Heating element material: NR  Heating element temperature: NR  Resistance: NR  Power/wattage: NR  Battery type: NR  Flavor: Camel  Nicotine content: 18 mg/mL  PG/VG content: NR  Additional e-liquid notes: N/A  EC09  Device type: NR  Device brand and model: Premium PR111  Device purchase date: NR  Additional type/brand notes: The EC was purchased online from Poland.  Voltage: NR  Heating element material: NR  Heating element temperature: NR  Resistance: NR  Power/wattage: NR  Battery type: NR  Flavor: Tobacco  Nicotine content: 16 mg/mL  PG/VG content: NR  Additional e-liquid notes: N/A  EC10  Device type: NR  Device brand and model: Ecis 510  Device purchase date: NR  Additional type/brand notes: The EC was purchased online from Poland.  Voltage: NR  Heating element material: NR  Heating element temperature: NR  Resistance: NR  Power/wattage: NR  Battery type: NR  Flavor: Menthol  Nicotine content: 11 mg/mL  PG/VG content: NR  Additional e-liquid notes: N/A  EC11  Device type: NR  Device brand and model: Dekang Pen  Device purchase date: NR  Additional type/brand notes: The EC was purchased online from Poland.  Voltage: NR  Heating element material: NR  Heating element temperature: NR  Resistance: NR  Power/wattage: NR  Battery type: NR  Flavor: Regular  Nicotine content: 18 mg/mL  PG/VG content: NR  Additional e-liquid notes: N/A  EC12  Device type: NR  Device brand and model: Intellicig Evolution  Device purchase date: NR  Additional type/brand notes: The EC was purchased online from the UK.  Voltage: NR  Heating element material: NR  Heating element temperature: NR  Resistance: NR  Power/wattage: NR  Battery type: NR  Flavor: Regular  Nicotine content: 8 mg/mL  PG/VG content: NR  Additional e-liquid notes: N/A | Number of devices  N=12  Number of samples  N=NR  Number of replicates  N=3 (each EC was tested 3 times on 3 following days after batteries were recharged during nights)  Control/comparator  Blank  Puffing machine  Palaczbot smoking machine  Puffing sets  N=10 of 15 puffs  Puffing regimen  Method: Based on results of inhalation topography measurement among 10 regular EC users  Puff duration: 1.8 sec  Intervals between puffs: 10 sec  Puff volume: 70 mL  Number of puffs: 150 (10 series of 15 puffs)  Puff profile: NR  Air flow speed: NR  Aerosol Collection Method  Smoke was extracted from vapor to the liquid phase using 2 gas-washing bottles with 100 mL of methanol. 20 mL of methanol solution was collected and condensed in vacuum evaporator.  Analytical method(s)  UPLC-MS | **Levels of nitrosamines, Mean±SEM**  **NNK (ng/150 puffs):**  EC01: 2.0±2.0  EC02: 3.6±1.8  EC03: 3.5±1.8  EC04: ND  EC05: ND  EC06: 1.1±1.1  EC07: 21.1±6.3  EC08: 4.6±0.4  EC09: 28.3±13.2  EC10: 2.1±2.1  EC11: 13.0±1.4  EC12: ND  LOD: 137 pg/150 puffs  LOQ: ND  EC Devices: 0.00011–0.00283 µg/15 puffs  Cigarettes*: 0.012–0.11 µg in mainstream smoke  Blank: ND  **NNN (ng/150 puffs):**  EC01: ND  EC02: 2.7±2.2  EC03: 0.8±0.8  EC04: ND  EC05: ND  EC06: 0.9±0.4  EC07: 4.3±2.4  EC08: 1.9±0.3  EC09: 1.2±0.6  EC10: 2.0±1.1  EC11: 3.2±0.6  EC12: 1.3±0.1  LOD: 46 pg/150 puffs  LOQ: ND  EC Devices: 0.00008–0.00043 µg/15 puffs  Cigarettes*: 0.005–0.19 µg in mainstream smoke  Blank: ND  ** Data extracted from Counts et al (2005).* |
| Jin et al. (2022)  Country  US  Laboratory site  US  Source of study funding  All authors were employees of Altria Client Services LLC.; study method was developed and validated internally in lab at Altria Client Services LLC. | EC01  Device type: Cig-a-Like  Device brand and model: NR  Device purchase date: NR  Additional type/brand notes: Rechargeable with disposable prefilled cartridges that were available in the US market in 2017.  Voltage: NR  Heating element material: NR  Heating element temperature: NR  Resistance: 3.4 Ω  Power/wattage: Low power  Battery type: NR  Flavor: NR  Nicotine content: 2.14%  PG/VG content: 33.4%/53.6% (w/w)  Additional e-liquid notes:  pH: 8.43  EC02  Device type: Cig-a-Like  Device brand and model: NR  Device purchase date: NR  Additional type/brand notes: Rechargeable with disposable prefilled cartridges that were available in the US market in 2017.  Voltage: NR  Heating element material: NR  Heating element temperature: NR  Resistance: 3.4 Ω  Power/wattage: Low power  Battery type: NR  Flavor: NR  Nicotine content: 1.70%  PG/VG content: ND/85.2% (w/w)  Additional e-liquid notes:  pH: 8.78  EC03  Device type: Cig-a-Like  Device brand and model: NR  Device purchase date: NR  Additional type/brand notes: Rechargeable with disposable prefilled cartridges that were available in the US market in 2017.  Voltage: NR  Heating element material: NR  Heating element temperature: NR  Resistance: 3.4 Ω  Power/wattage: Low power  Battery type: NR  Flavor: NR  Nicotine content: 2.17%  PG/VG content: 35.0%/54.2% (w/w)  Additional e-liquid notes:  pH: 8.65  EC04  Device type: Cig-a-Like  Device brand and model: MarkTenXL  Device purchase date: NR  Additional type/brand notes: Rechargeable with disposable prefilled cartridges that were available in the US market in 2017.  Voltage: NR  Heating element material: NR  Heating element temperature: NR  Resistance: 3.6 Ω  Power/wattage: Low power  Battery type: NR  Flavor: Menthol  Nicotine content: 3.34%  PG/VG content: 49.6%/33.2% (w/w)  Additional e-liquid notes:  pH: 9.48  EC05  Device type: Cig-a-Like  Device brand and model: MarkTenXL  Device purchase date: NR  Additional type/brand notes: Rechargeable with disposable prefilled cartridges that were available in the US market in 2017.  Voltage: NR  Heating element material: NR  Heating element temperature: NR  Resistance: 3.5 Ω  Power/wattage: Low power  Battery type: NR  Flavor: Classic  Nicotine content: 3.36%  PG/VG content: 23.8%/56.1% (w/w)  Additional e-liquid notes:  pH: 8.15  EC06  Device type: Cig-a-Like  Device brand and model: NR  Device purchase date: NR  Additional type/brand notes: Rechargeable with disposable prefilled cartridges that were available in the US market in 2017.  Voltage: NR  Heating element material: NR  Heating element temperature: NR  Resistance: 2.8 Ω  Power/wattage: Low power  Battery type: NR  Flavor: NR  Nicotine content: 4.56%  PG/VG content:  Product F: 16.9%/66.8% (w/w)  Additional e-liquid notes:  pH: 7.11  EC07  Device type: Cig-a-Like  Device brand and model: NR  Device purchase date: NR  Additional type/brand notes: Rechargeable with disposable prefilled cartridges that were available in the US market in 2017.  Voltage: NR  Heating element material: NR  Heating element temperature: NR  Resistance: 2.3 Ω  Power/wattage: NR  Battery type: Low power  Flavor: Menthol  Nicotine content: 4.43%  PG/VG content: 24.2%/59.0% (w/w)  Additional e-liquid notes:  pH: 7.44  EC08  Device type: Cig-a-Like  Device brand and model: NR  Device purchase date: NR  Additional type/brand notes: Rechargeable with disposable prefilled cartridges that were available in the US market in 2017.  Voltage: NR  Heating element material: NR  Heating element temperature: NR  Resistance: 2.1 Ω  Power/wattage: Low power  Battery type: NR  Flavor: NR  Nicotine content: 4.33%  PG/VG content: 24.1%/59.2% (w/w)  Additional e-liquid notes:  pH: 7.48 | Number of devices  N=8  Number of samples  N=NR  Number of replicates  N=4 per EC  Control/comparator  Commercial cigarette  Blank  Puffing machine  Linear 20-port puffing machine (Cerulean SM450)  Puffing sets  N=1  Puffing regimen  Method: Similar to CORESTA Method No.81  Puff duration: 5 sec  Intervals between puffs: 30 sec  Puff volume: 55 mL  Number of puffs: 150 (except for 50 puffs for MarkTen XL in the experiment of nitrite fortification to e-liquid)  Puff profile: Square wave  Air flow speed: NR  Aerosol Collection Method  Aerosols were collected on 44 mm fiberglass Cambridge filter pads.  Analytical method(s)  UPLC-MS/MS | **Levels of nitrosamines, Mean**  **NNK (ng/g)**  EC01: 0.40 (0.0015 ng/puff)  EC02: 4.46 (0.021 ng/puff)  EC03: 0.90 (0.0045 ng/puff)  MarkTen XL (Menthol): 2.55 (0.011 ng/puff)  MarkTen XL (Classic): 1.98 (0.0098 ng/puff)  EC06: 2.28 (0.0083 ng/puff)  EC07: 13.3 (0.046 ng/puff)  EC08: 45.8 (0.16 ng/puff)  LOQ: 0.2 (0.002 ng/puff)  Blank: NR  **NNK (ng/EC or cigarette)**  EC01: 0.16  EC02: 2.33  EC03: 0.50  MarkTen XL (Menthol): 1.56  MarkTen XL (Classic): 1.44  EC06: 1.14  EC07: 6.41  EC08: 22.5  Cigarette (ISO average)*: 55  Cigarette (Intense average)*: 122  Blank: NR  *Nitrite added to MarkTen XL e-liquid†:*  Nitrite (0 µg/g): <0.37  Nitrite (2 µg/g): 12.0±1.5  Nitrite (10 µg/g): 53.8±7.4  LOQ: 0.2  Blank: NR  **NNN (ng/g)**  EC01: ND  EC02: 30.3 (0.15 ng/puff)  EC03: ND  MarkTen XL (Menthol): 3.03 (0.013 ng/puff)  MarkTen XL (Classic): 0.65 (0.0032 ng/puff)  EC06: 0.79 (0.003 ng/puff)  EC07: 3.45 (0.012 ng/puff)  EC08: 26.3 (0.082 ng/puff)  LOQ: 0.4 (0.004 ng/puff)  Blank: NR  **NNN (ng/EC or cigarette)**  EC01: ND  EC02: 15.8  EC03: ND  MarkTen XL (Menthol): 1.84  MarkTen XL (Classic): 0.47  EC06: 0.42  EC07: 1.66  EC08: 11.7  Cigarette (ISO average)*: 85  Cigarette (Intense average)*: 189  Blank: NR  *Nitrite added to MarkTen XL e-liquid†:*  Nitrite (0 µg/g): <0.73  Nitrite (2 µg/g): 1.6±0.3  Nitrite (10 µg/g): 3.4±0.9  LOQ: 0.4  Blank: NR  **Data extracted from Edwards et al (2017).*  *† E-liquid composed of 82.5% (w/w) PG/GLY (50:50), 15% water, and 2.5% nicotine.* |
| Leigh et al. (2018)  Country  US  Laboratory site  US  Source of study funding  NCI of the NIH | EC01  Device type: NR  Device brand and model: MarkTen Device purchase date: NR  Additional type/brand notes:  N/A  Voltage: NR  Heating element material: NR  Heating element temperature: NR  Resistance: NR  Power/wattage: NR  Battery type: NR  Flavor: Tobacco  Nicotine content: 3.5%  PG/VG content: NR  Additional e-liquid notes: N/A | Number of devices  N=1  Number of samples  N=NR  Number of replicates  N=3 per product  Control/comparator  Marlboro Red 100 Cigarette  Blank  Puffing machine  Borgwaldt LX-1 smoking machine  Puffing sets  N=1  Puffing regimen  Method: Health Canada Intense  Puff duration: 2 sec  Intervals between puffs: 30 sec  Puff volume: 55 mL  Number of puffs: 55  Puff profile: NR  Air flow speed: NR  Aerosol Collection Method  Cambridge filters (44 mm) were used to capture the total particulate matter. Filters were spiked with deuterated internal standards and extracted using 20 mL 100 mM ammonium acetate.  Analytical method(s)  LC-MS/MS | **Levels of nitrosamines, Mean**  **NNK (ng/puff):**  MarkTen: <LOQ  Cigarette: ~6.604*  Blank: ND  LOQ: 0.5 ng/filter  **NNN (ng/puff):**  MarkTen: ~0.017*  Cigarette: ~14.230*  Blank: ND  LOQ: 0.5 ng/filter  ** Approximate values were derived from Figure 1 using an automated program.* |
| Margham et al. (2016)  Country  UK  Laboratory site  Canada  Source of study funding  BAT | EC01  Device type: Closed-modular system  Device brand and model: Vype ePen  Device purchase date: NR  Additional type/brand notes:  Consisted of 2 modules: a rechargeable battery section and a replaceable e-liquid containing cartridge (“cartomizer”); Had a removable mouthpiece and a screw connector for the cartomizer to connect to the battery section.  Voltage: 3.6 and 4 V (selectable by the user)  Heating element material: NiCr of 80% Ni and 20% Cr (silica transport wick)  Heating element temperature: NR  Resistance: 2.85 Ω  Power/wattage: NR  Battery type: USB-rechargeable; 650 mAh  Flavor: Blended Tobacco (<1%)  Nicotine content: 1.86%  PG/VG content: 25%/48.14% (w/w)  Additional e-liquid notes: N/A | Number of devices  N=1  Number of samples  N=1 product was sampled at a single point in time  Number of replicates  N=5 replicates of products sampled at 1 point in time  Control/comparator  Ky3R4F Kentucky Reference Cigarette  Blank  Puffing machine  NR  Puffing sets  N=2 blocks of 100 puffs each  Puffing regimen  Method: CORESTA Method No.81  Puff duration: 3 sec  Intervals between puffs: Puffs were taken twice per minute  Puff volume: 55 cm^3^  Number of puffs: 200  Puff profile: Rectangular  Air flow speed: NR  Aerosol Collection Method  Aerosols were collected on pads extracted with a 100 mM ammonium acetate solution.  Analytical method(s)  LC-MS/MS | **Levels of nitrosamines, Mean±SD**  **NNK (ng/collection):**  Vype ePen*:  Puffs 1-100: BDL  Puffs 101-200: NQ  Puffs 1-200: 0.01 ng/puff  LOD: 0.753  LOQ: 2.51  Cigarette: 282.67±24.17 ng/10.6 puffs (26.67 ng/puff)  LOD: 0.3  LOQ: 1.00  Blank:  Puffs 1-100: BDL  Puffs 101-200: NQ  Puffs 1-200: 0.016 ng/puff  **NNN (ng/collection):**  Vype ePen*:  Puffs 1-100: 5.16±0.56  Puffs 101-200: 5.61±1.03  Puffs 1-200: 0.054 ng/puff  LOD: 0.492  LOQ: 1.641  Cigarette: 264.67±22.2 ng/10.6 puffs (24.97 ng/puff)  LOD: 0.2  LOQ: 0.66  Blank:  Puffs 1-100: NQ  Puffs 101-200: 1.83±0.51  Puffs 1-200: 0.014 ng/puff  ** The EC was operated at the 3.6 V setting.* |
| Margham et al. (2021)  Country  UK  Laboratory site  UK & Canada  Source of study funding  BAT | EC01  Device type: NR  Device brand and model: Vype ePen2  Device purchase date: NR  Additional type/brand notes: Consisted of rechargeable battery and disposable e-liquid cartridge (1.58 mL).  Voltage: 3.5-3.7 V  Heating element material: NiCr of 80% Ni and 20% Cr (silica wick)  Heating element temperature: NR  Resistance: 2.85 Ω  Power/wattage: NR  Battery type: Micro-USB rechargeable  Flavor: Golden Tobacco, Dark Cherry, and Crisp Mint  Nicotine content: 1.86%  PG/VG content:  Golden Tobacco: 24.97%/48.14% (w/w)  Dark Cherry: 23.86%/48.14% (w/w)  Crisp Mint: 34.73%/37.64% (w/w)  Additional e-liquid notes: N/A | Number of devices  N=1  Number of samples  N=3 flavor variants of the EC  Number of replicates  N=5 replicates of products sampled at 1 point in time  Control/comparator  Ky3R4F Kentucky Reference Cigarette  Blank  Puffing machine  NR  Puffing sets  N=2 of 100 puffs each*  Puffing regimen  Method: CORESTA Method No.81 (ISO 20768:2018)  Puff duration: 3 sec  Intervals between puffs: Puffs were taken twice per minute  Puff volume: 55 mL  Number of puffs: 100 puffs  Puff profile: NR  Air flow speed: NR  Aerosol Collection Method  Aerosols were collected on Cambridge Filter pads extracted with a 100 mM ammonium acetate solution.  Analytical method(s)  LC-MS/MS  ** Emissions were collected and analyzed from 2 successive 100 puff blocks. Since no significant differences were found between the first and second 100-puff block, it was decided to analyze only the first 100.* | **Levels of nitrosamines, Mean±SD**  **NNK (ng/100 puffs):**  *EC01**  Crisp Mint: BDL (0.00376 ng/puff)  Dark Cherry: BDL (0.00376 ng/puff)  Golden Tobacco: BDL (0.00376 ng/puff)  LOD: 0.75  LOQ: 2.51  Blank: BDL (0.00376 ng/puff)  Cigarette: 105.84±3.95 ng/8.3 puffs (12.75 ng/puff)  LOD: 0.15  LOQ: 0.50  Blank: BDL (0.00836 ng/puff)  **NNN (ng/100 puffs)**:  *EC01**  Crisp Mint: BDL (0.00246 ng/puff)  Dark Cherry: BDL (0.00246 ng/puff)  Golden Tobacco: BDL (0.00246 ng/puff)  LOD: 0.49  LOQ: 1.64  Blank: BDL (0.00246 ng/puff)  Cigarette: 106.96±5.60 ng/8.3 puffs (12.89 ng/puff)  LOD: 0.10  LOQ: 0.33  Blank: BDL (0.00547 ng/puff)  ** The EC was operated at the 3.7 V setting (high power).* |
| Nicol et al. (2020)  Country  UK  Laboratory site  Canada  Source of study funding  BAT | EC01  Device type: e-pen  Device brand and model: NR  Device purchase date: NR  Additional type/brand notes:  Rechargeable battery and disposable cartridge (1.95 mL).  The EC was factory-sampled.  Voltage: NR  Heating element material: Stainless-steel mesh  Heating element temperature: NR  Resistance: NR  Power/wattage: 10 W  Battery type: Rechargeable  Flavor: Twilight Tobacco  Nicotine content: 5 mg/mL (0.43% w/w)  PG/VG content: 36%/62.6% (w/w)  Additional e-liquid notes: N/A | Number of devices  N=1  Number of samples  N=1 flavor variant sampled from the factory at a single point in time  Number of replicates  N=5 per sample  Control/comparator  1R6F Kentucky Reference Cigarette  Blank  Puffing machine  NR  Puffing sets  N=1  Puffing regimen  Method: CORESTA Method No.81 (ISO 20768:2018)  Puff duration: 3 sec  Intervals between puffs: 30 sec  Puff volume: 55 mL  Number of puffs: 50  Puff profile: Square wave  Air flow speed: NR  Aerosol Collection Method  Aerosols were collected on pads extracted with a 100 mM ammonium acetate solution.  Analytical method(s)  LC-MS/MS | **Levels of nitrosamines, Mean±SD**  **NNK (ng/50 puffs)**  EC01: BDL  LOD: 0.753  LOQ: 2.51  Blank: BDL  Cigarette*: 218.4±16.6 ng/cigarette  LOD: 0.251 ng/cigarette  LOQ: 0.836 ng/cigarette  Blank: BDL  **NNN (ng/50 puffs)**  EC01: BDL  LOD: 0.492  LOQ: 1.64  Blank: BDL  Cigarette*: 178.13±7.14 ng/cigarette  LOD: 0.164 ng/cigarette  LOQ: 0.547 ng/cigarette  Blank: BDL  **Cigarette: 9.1±0.3 puffs* |
| Pinto et al. (2022)  Country  UK  Laboratory site  Canada  Source of study funding  BAT | EC01  Device type: Pod (4^th^ generation)  Device brand and model: Vype ePod1.0  Device purchase date: NR  Additional type/brand notes:  Consisted of a metallic outer device case, a printed circuit board to control the device, a lithium-ion rechargeable battery, and a cartridge.  Each pod was pre-filled with  Vype e-liquid (1.9 mL) and was magnetically attached to the device.  The EC was designed with  innovative ceramic wick-based technology.  Voltage: 2.2 to 3.1 V (not adjustable by the user)  Heating element material: NiCr (ceramic wick material)  Heating element temperature: <350°C (low)  Resistance: 0.8-1.4 Ω  Power/wattage: 6.5±0.5 W  Battery type: Lithium-ion rechargeable; 350 mAh  Flavor: Berry Blast  Nicotine content: 57 and 18 mg/mL  PG/VG content: 50%/50% (w/w)  Additional e-liquid notes:  Two e-liquid samples; Berry Blast 57 mg/mL contained lactic acid and Berry Blast 18 mg/mL contained BA. | Number of devices  N=1  Number of samples  N=2 flavored e-liquids  Number of replicates  N=5 independent replicates per sample  Control/comparator  1R6F Kentucky Reference cigarette  Blank  Puffing machine  Rotary or a linear smoking machine  Puffing sets  N=1  Puffing regimen  Method: CORESTA Method No.81 (ISO 20768:2018)  Puff duration: 3 sec  Intervals between puffs: 30 sec  Puff volume: 55 mL  Number of puffs: 50  Puff profile: Rectangular  Air flow speed: NR  Aerosol Collection Method  Aerosols were collected on pads extracted with ammonium acetate solution.  Analytical method(s)  LC-MS/MS | **Levels of nitrosamines, Mean±SD**  **NNK (ng/puff):**  *EC01*  Berry Blast (18 mg/mL): BDL  Berry Blast (57 mg/mL): BDL  LOD: 0.75 ng/50 puffs  LOQ: 2.51 ng/50 puffs  Cigarette*: 20.9±0.70 ng/cigarette  LOD: 0.25 ng/cigarette  LOQ: 0.84 ng/cigarette  Blank: <LOD  **NNN (ng/puff):**  Berry Blast (18 mg/mL): BDL  Berry Blast (57 mg/mL): BDL  LOD: 0.49 ng/50 puffs  LOQ: 1.64 ng/50 puffs  Cigarette*: 25.8±0.90 ng/cigarette  LOD: 0.16 ng/cigarette  LOQ: 0.55 ng/cigarette  Blank: BDL  ** Cigarette: ~9 puffs* |
| Poynton et al. (2017)  Country  UK  Laboratory site  Canada  Source of study funding  NR; Services were provided by the Analytical Development Center and Product Stewardship Service of Global R&D of BAT | EC01  Device type: Closed-modular system  Device brand and model: ePen (known as Vype ePen I)  Device purchase date: NR  Additional type/brand notes: Consisted of rechargeable battery and replaceable cartomizer with e-liquid tank and atomizer.  The EC was factory-sampled.  Voltage: 3.6 and 4.0 V  Heating element material: NR  Heating element temperature: NR  Resistance: NR  Power/wattage: NR  Battery type: USB-rechargeable; 650 mAh  Flavor: Blended Tobacco  Nicotine content: NR  PG/VG content: NR  Additional e-liquid notes: N/A | Number of devices  N=1  Number of samples  N=1 e-liquid flavor variant  Number of replicates  N=5 independent replicates sampled at one point in time  Control/comparator  Ky3R4F Kentucky Reference Cigarette  Blank  Puffing machine  NR  Puffing sets  N=2 (100 puffs per set)  Puffing regimen  Method: CORESTA  Puff duration: 3 sec  Intervals between puffs: 30 sec  Puff volume: 55 mL  Number of puffs: 100 puffs per set  Puff profile: Rectangular flow  Air flow speed: NR  Aerosol Collection Method  Aerosol were collected using Cambridge filter pads or impingers.  Analytical method(s)  LC-MS/MS | **Levels of nitrosamines, Mean±SD**  **NNK (ng/100 puffs):**  ePen*:  Puffs 1-100: NQ  Puffs 101-200: NQ  Cigarette^†^: 283±24 ng/cigarette  LOD: 0.30 ng/cigarette  LOQ: 1.00 ng/cigarette  Blank:  Puffs 1-100: NR  Puffs 101-200: NR  **NNN (ng/100 puffs):**  ePen*:  Puffs 1-100: NQ  Puffs 101-200: NQ  Cigarette^†^: 265±22 ng/cigarette  LOD: 0.20 ng/cigarette  LOQ: 0.66 ng/cigarette  Blank:  Puffs 1-100: NR  Puffs 101-200: NR  ** The device voltage was set at 3.6 V.*  *† Cigarette: ~9 puffs* |
| Rudd et al. (2020)  Country  UK  Laboratory site  UK  Source of study funding  Imperial Brands PLC was the source of funding; No external sources of funding were involved. | EC01  Device type: Pod  Device brand and model: Myblu^TM^  Device purchase date: NR  Additional type/brand notes:  Closed pod-system consisting of a rechargeable battery and a replaceable e-liquid containing pod.  EC was purchased from UK retailers.  Voltage: NR  Heating element material: NR  Heating element temperature: NR  Resistance: 1.3 Ω  Power/wattage: NR  Battery type: Rechargeable; 350 mAh  Flavor: Tobacco  Nicotine content: 1.6%  PG/VG content: NR  Additional e-liquid notes: N/A | Number of devices  N=1  Number of samples  N=1 flavor  Number of replicates  N=3 replicates for each 50-puff block  Control/comparator  3R4F Kentucky Reference Cigarette  Blank  Puffing machine  Borgwaldt linear smoking machine LMC4  Puffing sets  N=3 separate 50-puff blocks  Puffing regimen  Method: CORESTA Method No.81  Puff duration: 3 sec  Intervals between puffs: 30 sec  Puff volume: 55 mL  Number of puffs: 150  Puff profile: Square wave  Air flow speed: NR  Aerosol Collection Method  Aerosols were collected on Cambridge filter pads, which were transferred to a 50 mL Erlenmeyer flask and extracted in 20 mL MeOH:H_2_O (1:1). An automated shaker was used.  Analytical method(s)  LC-MS/MS | **Levels of nitrosamines, Mean±SD**  **NNK (ng/collection*):**  Myblu: BDL  LOD: 6.7  LOQ: 20.0  Cigarette: 237.87±9.78  LOD: 2.7  LOQ: 8.0  Blank: NR  **NNN (ng/collection*):**  Myblu: BDL  LOD: 6.7  LOQ: 20.0  Cigarette: 363.97±8.45  LOD: 2.7  LOQ: 8.0  Blank: NR  **EC: 150 puffs*  *Cigarette: ~10 puffs* |
| Tayyarah et al. (2014)  Country  US  Laboratory site  US  Source of study funding  NR; Authors were employees of Lorillard Tobacco Company, which manufactured the ECs tested in the study. | EC01  Device type: Disposable  Device brand and model: blu  Device purchase date: NR  Additional type/brand notes:  ECs were manufactured by blu eCigs  Voltage: NR  Heating element material: NR  Heating element temperature: NR  Resistance: NR  Power/wattage: NR  Battery type: NR  Flavor: Classic Tobacco (7%) and Magnificent Menthol (5%)  Nicotine content: 24 mg/unit  PG/VG content:  blu CTD: 82% glycerin  blu MMD: 75% glycerin  Additional e-liquid notes: N/A  EC02  Device type: Rechargeable  Device brand and model: blu  Device purchase date: NR  Additional type/brand notes:  ECs were manufactured by blu eCigs.  Voltage: NR  Heating element material: NR  Heating element temperature: NR  Resistance: NR  Power/wattage: NR  Battery type: Rechargeable  Flavor: Cherry Crush (7%)  Nicotine content: 16 mg/unit  PG/VG content: 77% glycerin  Additional e-liquid notes: N/A  EC03  Device type: Rechargeable  Device brand and model: SKYCIG  Device purchase date: NR  Additional type/brand notes:  ECs were manufactured by SKYCIG.  Voltage: NR  Heating element material: NR  Heating element temperature: NR  Resistance: NR  Power/wattage: NR  Battery type: Rechargeable  Flavor: Classic Tobacco Bold (1%) and Crown Menthol Bold (4%)  Nicotine content: 18 mg/unit  PG/VG content:  SKYCIG CTB: 67%/24%  SKYCIG CMB: 66%/21%  Additional e-liquid notes: N/A | Number of devices  N=5 (2 disposable and 3 rechargeable)  Number of samples  N=NR  Number of replicates  N=3 to 5 replicates for each sample  Control/comparator  Marlboro Gold Box cigarettes  L&B Original and Menthol  Blank  Puffing machine  NR  Puffing sets  N=1  Puffing regimen  Method: Health Canada Test Method T-115  Puff duration: NR  Intervals between puffs: Puffs were taken twice in 1 min  Puff volume: 55 mL  Number of puffs: 99  Puff profile: NR  Air flow speed: NR  Aerosol Collection Method  Aerosol particulate was collected onto 44 mm GFFP extracted with 20 mL of 5 mM aqueous ammonium.  Analytical method(s)  LC-MS/MS | **Levels of nitrosamines, Mean±SD**  **NNK (ng/puff):**  blu CTD: BDL  blu MMD: BDL  blu CCH: BDL  LOD: 0.02  LOQ: 0.06  SKYCIG CTB: BDL  SKYCIG CBM: BDL  LOD: 0.03  LOQ: 0.10  Marlboro cigarette: 14.7±0.9  LOD: 0.02  LOQ: 0.06  L&B Original cigarette: 10.12±1.34  L&B Menthol cigarette: 7.36±1.39  LOD: 0.03  LOQ: 0.10  Blank: <LOD  **NNN (ng/puff):**  blu CTD: BDL  blu MMD: BDL  blu CCH: BDL  LOD: 0.02  LOQ: 0.06  SKYCIG CTB: BDL  SKYCIG CMB: BDL  LOD: 0.03  LOQ: 0.10  Marlboro cigarette: 19.5±1.5  LOD: 0.02  LOQ: 0.06  L&B Original cigarette: 7.93±0.73  L&B Menthol cigarette: 7.50±0.83  LOD: 0.03  LOQ: 0.10  Blank: <LOD |

Abbreviations: Ω = Ohm; µg = Microgram(s); BA = Benzoic Acid; BAT = British American Tobacco Investments Ltd.; BT = Blended Tabacco; CCH = Cherry Crush, Premium, High Strength; cm^3^ = Cubic centimeter; CMB = Crown Menthol Bold; CORESTA = Cooperation Centre for Scientific Research Relative to Tobacco; Cr = Chromium; CTB = Classic Tobacco Bold; CTD = Classic Tobacco Disposable; EC = Electronic Cigarette; ENDS = Electronic Nicotine Delivery Systems; ESI = Electrospray Ionization; g = Gram(s); GC = Gas Chromatography; GFFP = Glass Fiber Filter Pad; H_2_O = Water; ISO = International Organization for Standardization; L&B = Lambert & Butler; LC = Liquid Chromatography; LOD = Limit of Detection; LOQ = Limit of Quantification; mAh = Milliampere-hour; MB = Master Blend; MEIN = Ministry of Science and Higher Education of Poland; MeOH = Menthol; mg = Milligram(s); mL = Milliliter(s); mm = Millimeter(s); mM = Millimolar; MMD = Magnificent Menthol Disposable; MS/MS = Tandem Mass Spectrometry; N/A = Not Applicable; NCI = National Cancer Institute; ND = Not detected; ng = Nanogram(s); Ni = Nickel; NiCr = Nichrome; NiFe = Nickel-Iron; NIH = National Institutes of Health; NNK = 4-(Methylnitrosamino)-1-(3-pyridyl)-1-butanone; NNN = N’-Nitrosonornicotine; NQ = Not Quantifiable; NR = Not Reported; PG = Propylene Glycol; PLC = Public Limited Company; SD = Standard Deviation; sec = Second(s); UK = United Kingdom; US = United States; USB = Universal Serial Bus; V = Volt; VG = Vegetable Glycerin; W = Watt; w/w = Weight/weight.
