# Supplementary material for: Tobacco-specific nitrosamine exposure from electronic cigarettes versus combustible cigarettes: an ad hoc analysis within a systematic review of emission studies
Source: Front Oncol. 2025 Dec 12;15:1729107. doi: 10.3389/fonc.2025.1729107 (PMC12740905; doi:10.3389/fonc.2025.1729107)
Supplement: Supplementary file 2 [file Table2.docx]

**Table S2. Quality assessment of Studies Included (QualSyst Tool)**

| # | Criteria | Cook  et al.  (2024) | Chen  et al.  (2021) | Cunningham  et al.  (2020) | Goniewicz  et al.  (2014) | Jin  et al.  (2022) | Leigh  et al.  (2018) | Margham  et al.  (2016) | Margham  et al.  (2021) | Nicol  et al.  (2020) | Pinto  et al.  (2022) | Poynton  et al.  (2017) | Rudd  et al.  (2020) | Tayyarah  et al.  (2014) |
| --- | --- | --- | --- | --- | --- | --- | --- | --- | --- | --- | --- | --- | --- | --- |
| 1 | **Question/Objective Suffciently Described** | 2 | 2 | 2 | 2 | 2 | 2 | 2 | 2 | 2 | 2 | 2 | 2 | 2 |
| 2 | **Study Design Evident and Appropriate** | 2 | 2 | 2 | 2 | 2 | 2 | 2 | 2 | 2 | 2 | 2 | 2 | 2 |
|  | **Method of subject/comparison group selection or source of information/input variables described and appropriate?** |  |  |  |  |  |  |  |  |  |  |  |  |  |
| 3 | a) Device characteristics reported | 1 | 0 | 1 | 0 | 1 | 0 | 1 | 2 | 1 | 2 | 1 | 1 | 1 |
| 4 | b) E-liquid characteristics reported | 1 | 1 | 2 | 1 | 1 | 1 | 2 | 2 | 2 | 2 | 0 | 1 | 2 |
| 5 | **Subject (and comparison group, if applicable) characteristics sufficiently described?** |  |  |  |  |  |  |  |  |  |  |  |  |  |
|  | a) Overall Sample size and for each group are reported | 2 | 2 | 2 | 1 | 1 | 1 | 2 | 2 | 2 | 2 | 2 | 2 | 1 |
| 6 | **If interventional and random allocation was possible, was it described?** | N/A | N/A | N/A | N/A | N/A | N/A | N/A | N/A | N/A | N/A | N/A | N/A | N/A |
| 7 | **If interventional and blinding of investigators was possible, was it reported?** | N/A | N/A | N/A | N/A | N/A | N/A | N/A | N/A | N/A | N/A | N/A | N/A | N/A |
| 8 | **If interventional and blinding of subjects was possible, was it reported?** | N/A | N/A | N/A | N/A | N/A | N/A | N/A | N/A | N/A | N/A | N/A | N/A | N/A |
|  | **Outcome and (if applicable) exposure measure(s) well defined and robust to measurement bias? Means of assessment reported?** |  |  |  |  |  |  |  |  |  |  |  |  |  |
| 9 | a) Assessment of background correction reported | 1 | 2 | 2 | 2 | 1 | 1 | 2 | 2 | 2 | 2 | 2 | 1 | 2 |
| 10 | b) Laboratory quality control method reported (e.g. inter-assay coefficient of variation) | 1 | 2 | 0 | 1 | 1 | 0 | 1 | 1 | 1 | 1 | 1 | 0 | 1 |
| 11 | c) Puffing regime was appropriate to device | 2 | 2 | 2 | 1 | 2 | 2 | 2 | 2 | 2 | 2 | 2 | 2 | 1 |
| 12 | **Sample size appropriate?** | N/A | N/A | N/A | N/A | N/A | N/A | N/A | N/A | N/A | N/A | N/A | N/A | N/A |
|  | **Analytic methods described/justified and appropriate?** |  |  |  |  |  |  |  |  |  |  |  |  |  |
| 13 | a) Method for derivatization reported | N/A | N/A | N/A | N/A | N/A | N/A | N/A | N/A | N/A | N/A | N/A | N/A | N/A |
| 14 | b) Method for aerosol collection reported | 2 | 2 | 2 | 2 | 2 | 2 | 2 | 1 | 2 | 2 | 0 | 2 | 2 |
| 15 | c) Method for aerosol analyses reported | 2 | 2 | 2 | 2 | 2 | 2 | 2 | 1 | 2 | 2 | 1 | 2 | 2 |
| 16 | **Some estimate of variance is reported for the main results?** | 2 | 0 | 2 | 2 | 1 | 1 | 2 | 2 | 2 | 2 | 2 | 2 | 2 |
| 17 | **Controlled for confounding?** | N/A | N/A | N/A | N/A | N/A | N/A | N/A | N/A | N/A | N/A | N/A | N/A | N/A |
|  | **Results reported in sufficient detail?** |  |  |  |  |  |  |  |  |  |  |  |  |  |
| 18 | a) Limits of detection (quantification) reported | 2 | 2 | 2 | 1 | 1 | 1 | 2 | 2 | 2 | 2 | 1 | 2 | 2 |
| 19 | b) Measures of central tendency reported (Means, Median, Geometric mean) | 2 | 2 | 2 | 2 | 1 | 1 | 2 | 2 | 2 | 2 | 2 | 2 | 2 |
| 20 | c) Were the chemical forms of reported emissions adequately described for interpretation? This includes speciation of metals and/or identification and quantification of individual carbonyl compounds. | N/A | N/A | N/A | N/A | N/A | N/A | N/A | N/A | N/A | N/A | N/A | N/A | N/A |
| 21 | **Conclusions supported by the results?** | 2 | 2 | 2 | 2 | 2 | 2 | 2 | 2 | 2 | 2 | 2 | 2 | 2 |
|  |  |  |  |  |  |  |  |  |  |  |  |  |  |  |
| Total Sum | | 24 | 23 | 25 | 21 | 20 | 18 | 26 | 25 | 26 | 27 | 20 | 23 | 24 |
| Highest Possible Sum | | 28 | 28 | 28 | 28 | 28 | 28 | 28 | 28 | 28 | 28 | 28 | 28 | 28 |
| Summary Score | | **0.86** | **0.82** | **0.89** | **0.75** | **0.71** | **0.64** | **0.93** | **0.89** | **0.93** | **0.96** | **0.71** | **0.82** | **0.86** |
| Quality Assessment | | **S** | **S** | **S** | **G** | **G** | **A** | **S** | **S** | **S** | **S** | **G** | **S** | **S** |

Scoring Legend: N/A= Not Applicable; 0= Criteria not reported/conducted; 1= Criteria partially reported/conducted; 2= Criteria fulfilled

Quality Assessment Legend: Strong (S) = Summary Score of > 0.80; Good (G)= Summary Score of 0.71 - 0.79; Adequate (A)= Summary Score of 0.50 - 0.70; Limited (L)= Summary Score of < 0.50
